# Profile Study on Different Patterns in PMMA Bilayer Resist Configurations Under 20 keV Electron Beam Exposure

**DOI:** 10.3390/nano16201311

**Published:** 2026-10-16

**Authors:** Ivan Kostic, Evgeniy Manoilov, Anna Bencurova, Elena Koleva, Robert Andok, Katia Vutova

**Affiliations:** 1 Institute of Informatics, Slovak Academy of Sciences, Dubravska Cesta 9, 845 07 Bratislava, Slovakia; anna.bencurova@savba.sk (A.B.); robert.andok@savba.sk (R.A.); 2 Institute of Electronics, Bulgarian Academy of Sciences, 72 Tsarigradsko Chaussee Blvd., 1784 Sofia, Bulgaria; evgeniy.manoilov@ie.bas.bg (E.M.); eligeorg@uctm.edu (E.K.); 3 Faculty of Chemical and Systems Engineering, University of Chemical Technology and Metallurgy, 8, Kliment Ohridski Blvd., 1756 Sofia, Bulgaria

**Keywords:** electron beam lithography, bilayer PMMA, resist profile, sidewall geometry, empirical modelling, exposure dose optimization

## Abstract

Electron beam lithography (EBL) using bilayer resists is influenced by electron scattering, proximity effects, resist thickness, pattern density, and exposure dose. Optimization based only on the top-view critical dimension does not capture linewidth variation through the developed resist thickness. This study presents an experimental and theoretical approach to model and optimize the depth-dependent sidewall geometry of bilayer PMMA exposed by a variable-shaped-beam EBL at 20 keV. Bilayers consisting of 950 PMMA A2 and 495 PMMA A6, with total thicknesses of 300 and 550 nm, on SiO_2_/Si substrates were fabricated. We investigated isolated lines and line gratings with nominal line/space (L/S) dimensions of 100/500 and 100/700 nm over selected exposure dose ranges. Cross-sectional SEM measurements at five resist depths were used to develop regression models describing linewidth as a function of the exposure dose and resist depth. Two models for mean values and standard deviations were constructed for each of the considered experimental cases to account for external noise not included in the regression models, and the propagated uncertainties are quantified by Monte Carlo simulation. Four linewidth-difference criteria to identify doses producing near-vertical profile segments were introduced and applied. The results demonstrate that the selected optimum dose depends strongly on the bottom-layer thickness, pattern density, and the profile segment considered. The proposed approach provides a quantitative empirical method for predicting, analyzing, controlling, and optimizing EBL resist profiles, and can also be applied, with appropriate experimental calibration, to different resist systems, pattern geometries, exposure dose ranges, beam shot sizes, and acceleration voltages.

## 1. Introduction

Although Monte Carlo [1,2,3] and analytical methods [4,5,6] are widely used in electron beam lithography (EBL) to describe electron scattering, proximity effects, and energy deposition, many existing models treat exposure and resist development separately, use simplified single-layer resist descriptions, or focus mainly on the final two-dimensional critical dimension (CD). In [1], Monte Carlo simulation techniques were extended to the case of laterally distributed e-beams and the contribution of backscattered particles from the substrate was included in the spatial energy distribution. Monte Carlo modelling methods using single and multiple scattering algorithms were described, and a practical guide to using this technique is provided in [2], while the MOCASEL tool for 3D Monte Carlo simulation of EBL on topographic substrates, introducing the “electron cloud” method to reduce computation time was presented in [3]. A simple technique for calculating the proximity effect in EBL was presented in [4], while a method for proximity effect correction by equalizing the background dose was proposed in [5]. Methods for determining proximity effect correction parameters in EBL for 0.1 μm structures were investigated and several techniques for fitting multiple Gaussian expressions to the radial absorbed energy density were discussed in [6]. Quantitative approaches that directly link the spatial and depth-dependent energy deposited in the resist with dose-dependent development and the resulting three-dimensional sidewall geometry remain comparatively limited. This is particularly relevant for bilayer resist systems used in lift-off processes and for nanostructure fabrication on different substrates, including 2-dimensional materials such as WS_2_. Recent experimental studies have highlighted that optimizing bilayer PMMA resist stacks (such as 950-k atop 495-k) is essential for achieving reproducible undercut profiles and defect-free lift-off in sub-50 nm metallic structures [7,8].

EBL modelling generally involves several interconnected stages: electron transport and scattering, energy deposition, resist exposure, development, and formation of the final resist profile. Early Monte Carlo approaches showed that the deposited energy distribution in PMMA (polymethyl methacrylate) depends not only on the incident electron beam but also on electron backscattering from the substrate. Kyser and Pyle [9] developed a computer-based simulation framework that combined Monte Carlo electron transport with a resist-development model. Their lithography modelling system accounted for the beam voltage, resist thickness, substrate material, and pattern geometry, and enabled prediction of the developed resist profile as a function of the exposure and development conditions.

Subsequent approaches extended such modelling toward complete 3D resist structures. The ProBEAM/3D approach [10], for example, combined Monte Carlo simulation of electron scattering with the actual beam shape, dose distribution, and development model, demonstrating that the 3D resist profile can be predicted by first calculating the local deposited-energy distribution and subsequently applying a development model. Similar comprehensive approaches combined Monte Carlo point-spread functions with proximity-effect correction, exposure, processing, and development, allowing evaluation of the resulting structures not only by CD but also by sidewall angle and residual resist thickness. Fito et al. [11] further demonstrated the importance of coupling the electron transport with the polymer-development physics by modelling primary, secondary, and backscattered electrons and the resulting distribution of main-chain scission events in PMMA. Their comparison with experimental cross-sectional profiles showed that the PMMA profile cannot generally be described by a single exposure threshold. These concepts align with broader reviews of EBL simulation, which emphasize that exposure, energy deposition, development, and processing effects should be considered as interconnected stages of the lithographic process [12].

In conventional proximity-effect correction, the primary objective is usually to obtain the required lateral CD. However, structures with similar top-view CDs can exhibit substantially different widths at the bottom of the resist and, consequently, different sidewall angles. Therefore, agreement in the top CD does not necessarily indicate agreement in the complete lithographic profile. Experimental and simulation studies show that PMMA sidewall geometry depends strongly on the exposure dose, electron energy, and resist thickness. Andok et al. [13] investigated PMMA layers with thicknesses of 600 and 1300 nm at 30 keV and analyzed structure widths at different resist depths as a function of the exposure dose. Their results demonstrated the importance of considering the depth-dependent proximity effect when predicting and controlling thick-PMMA profiles. Related modelling approaches have also examined the relationship between the total dose, spatial dose distribution, development conditions, and sidewall shape, with the objective of obtaining either vertical or intentionally sloped/undercut profiles [14]. Recent modelling demonstrates that accounting for depth-dependent, non-uniform energy deposition in the positive resist ELP-20 is critical for accurate resist contrast determination in EBL [15]. By applying multidimensional optimization to a negative e-beam photoresist, researchers have improved the resolution, sidewall roughness, and reproducibility of nanostructures [16].

The situation becomes more complex in multilayer resist systems, where different layers may have different sensitivities, dissolution rates, and molecular characteristics. Consequently, the deposited-energy distribution and development response can vary with depth, changing linewidth and sidewall slope across the resist thickness. Nemec et al. [17] experimentally and numerically investigated bilayer and trilayer resist systems and analyzed linewidths at several depths. Monte Carlo calculations of the deposited energy were approximated using a double-Gaussian distribution characterized by the proximity-effect parameters α, β, and η. The results showed that the bottom-layer thickness can strongly influence the resulting sidewall geometry, with increased bottom-layer thickness producing profiles closer to vertical. This behaviour was mainly associated with long-range electron scattering and backscattering from the substrate. This depth response is further governed by the resist’s molecular weight and polydispersity, which dictate the local chain-scission rate and dissolution contrast throughout the PMMA stack [18].

A further step toward application-oriented modelling was reported by Bencurova et al. [19], who investigated the sidewall shape and proximity effect in a PMMA bilayer deposited on a thin WS_2_ film. This work provides an experimental basis for studying resist-profile formation in EBL structures intended for 2D-material applications. Empirical approaches and dose-assignment frameworks offer practical alternatives to computationally intensive 3D numerical models for determining viable lithographic process windows [20]. To improve resist profile prediction, a novel dose-assignment methodology that integrates a refined contrast curve and the point spread function was proposed [20]. Nevertheless, a quantitative model that simultaneously considers dose-dependent development and the resulting PMMA sidewall geometry remains insufficiently explored, particularly for bilayer systems and different resist/substrate configurations.

The present study extends our previous investigation of line-grating profiles in bilayer PMMA by considering five experimental cases comprising isolated lines and gratings, two total bilayer thicknesses, and two grating spacings. The aim of the present study is to develop an empirical modelling approach for predicting, analyzing, controlling, and optimizing developed resist profiles, obtained by EBL in different PMMA bilayer resist systems and structures (isolated lines and gratings). Regression models describing the developed linewidth as a function of the exposure dose and resist depth were developed. The nature of the variations and the adoption of the robust methodology for assessing the overall variation in the output were also considered, as was the accounting for the uncertainty arising from external noise and process parameter variability using a Monte Carlo simulation approach. Dual response surface models were constructed for the mean values and standard deviations of the developed profile linewidths. The models were calibrated using measurements on five experimental datasets obtained from a 20 keV VSB system, two PMMA bilayer thicknesses, a SiO_2_/Si substrate, and fixed development conditions. Application of the proposed procedure to another EBL process requires new cross-sectional measurements and model calibration.

## 2. Materials and Methods

We performed electron beam lithography (EBL) using a ZBA21 system (Vistec Electron Beam GmbH, Jena, Germany) operated at an acceleration voltage of 20 keV [21]. We employed a rectangular variable-shaped beam (VSB), providing a nominally homogeneous electron-energy distribution within a tolerance of 10%. In variable-shaped beam systems, shot-count approximation and edge dose assignment are critical to minimize line-edge roughness (LER) [22]. We set the beam current density to 800 µA/cm^2^, providing a suitable compromise between exposure throughput and pattern resolution.

For line-grating fabrication, we selected a nominal VSB shot size of 100 × 3000 nm^2^, with the 100 nm dimension defining the nominal exposed linewidth. Gratings were designed with nominal line-to-space (L/S) dimensions of 100/500 nm and 100/700 nm, corresponding to periods of 600 nm and 800 nm, respectively. The reduced nominal linewidth provided geometric precompensation for lateral broadening caused by the electron scattering, proximity effects, and resist development. With an appropriate exposure dose, these designs were intended to yield final developed L/S dimensions of 300/300 nm and 400/400 nm, respectively, while preserving the grating period.

**Figure 1:**
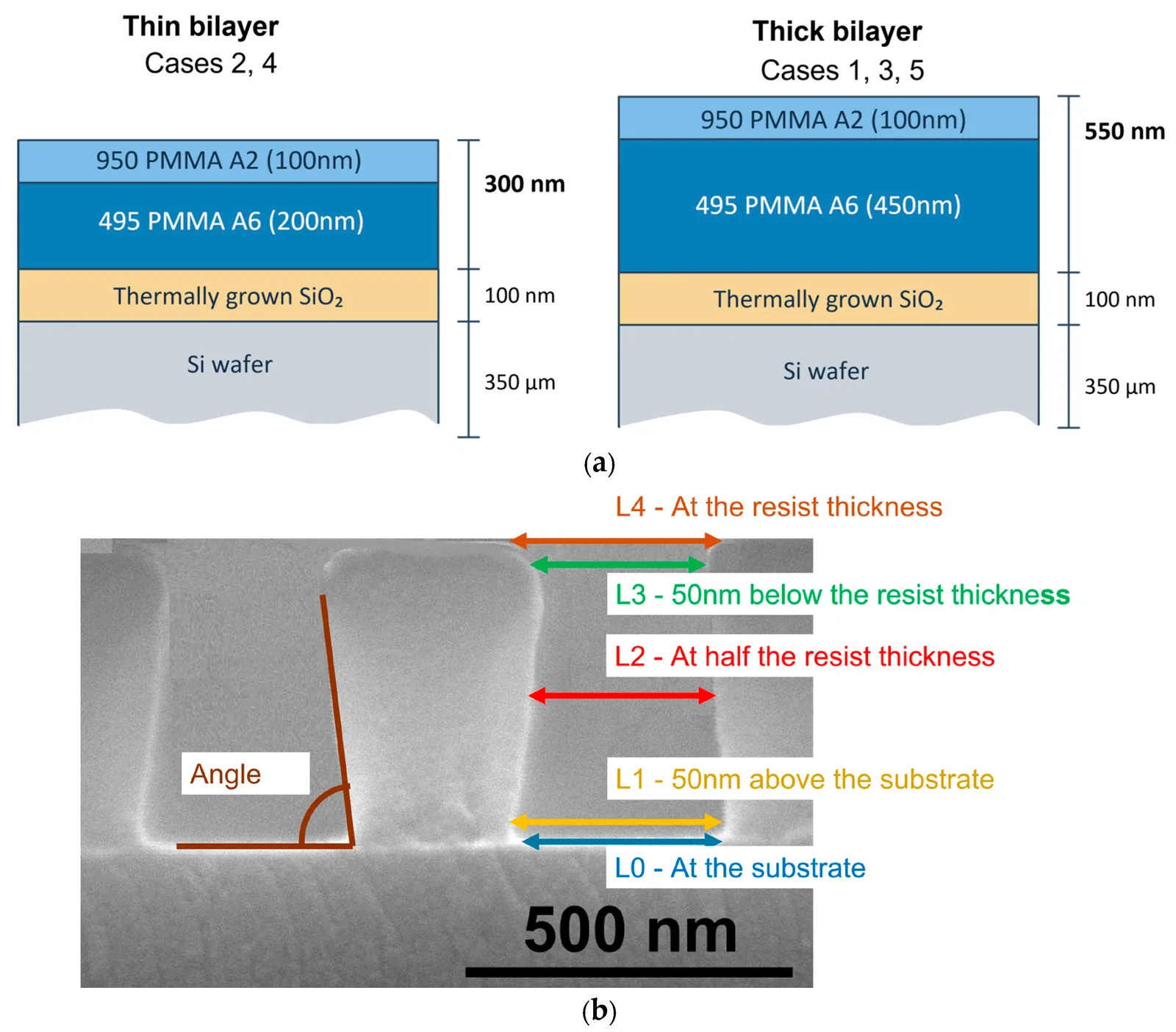
(**a**) Schematic of bilayer PMMA resist systems: Cases 2 and 4—100 nm 950 PMMA A2 top layer on a 200 nm 495 PMMA A6 bottom layer (total thickness: 300 nm); Cases 1, 3, and 5—100 nm 950 PMMA A2 top layer on a 450 nm 495 PMMA A6 bottom layer (total thickness: 550 nm). (**b**) Schematic of linewidth measurement levels across the resist profile depth.

We fabricated the lithographic patterns using a PMMA bilayer resist system with total thicknesses of 300 nm (Cases 2 and 4, denoted in figures and tables as N2 and N4) and 550 nm (Cases 1, 3, and 5, denoted as N1, N3, and N5). The bottom layer consisted of 495 PMMA A6 (495 k molecular weight, anisole solvent, 6 wt.% concentration; Kayaku Advanced Materials, Inc., Westborough, MA, USA), while the top layer consisted of 950 PMMA A2 (950 k molecular weight, anisole solvent, 2 wt.% concentration; Kayaku Advanced Materials). A schematic of the bilayer resist systems is shown in Figure 1a. The PMMA bilayer was deposited on a substrate consisting of a 100 nm thermally grown SiO_2_ layer on a 350 µm-thick Si wafer. Dielectric oxide layers can induce charging effects during EBL that narrow the available exposure dose window [23]; at 20 keV, the electron penetration depth readily traverses the thin 100 nm SiO_2_ layer into the silicon substrate, mitigating localized charge build-up [24].

We separately prebaked each PMMA layer on a hot plate at 170 °C for 5 min. The resulting resist thicknesses were measured using an Alpha-Step profilometer.

The test patterns comprised single lines with a nominal linewidth (LW) of 500 nm and line gratings with L/S ratios of 100/500 nm and 100/700 nm. We varied the exposure dose (D) from 360 µC/cm^2^ to 2120 µC/cm^2^. A single exposure was used for the complete PMMA bilayer rather than separate exposures of the top and bottom resist layers.

After exposure, we developed the samples in a single step using a methyl isobutyl ketone (MIBK)/isopropyl alcohol (IPA) mixture at a volume ratio of 1:3 [25]. This developer is compatible with both 950 PMMA A2 and 495 PMMA A6 resists. The development time was 60 s. We terminated development by rinsing the samples with IPA to remove the dissolved resist and suppress residual swelling effects.

For cross-sectional characterization, we coated the samples with a thin platinum layer and then cleaved them perpendicularly to the patterned lines. Resist linewidths were measured by scanning electron microscopy (SEM) using an FEG 250 system (FEI, USA) at 200,000×–300,000× magnification. Cross-sectional measurements were performed at five different levels along the resist thickness (Figure 1b): at the top surface of the resist (L4), at 50 nm below the top surface (L3), at the middle of the resist thickness (L2), at 50 nm above the substrate (L1), and at the resist/substrate interface (L0). The measurements were used to determine the depth-dependent resist sidewall profile and to provide experimental data for subsequent resist-profile simulations [26].

## 3. Results and Discussion

### 3.1. Experimental Results

In our experiment, we focused on a variable-shaped beam, which significantly increases the throughput compared with a conventional Gaussian spot beam. This is particularly advantageous for large-area, high-density fabrication. However, the VSB approach also introduces limitations, as the ZBA21 system has a minimum beam size of 100 nm and an electron energy of 20 keV. Consequently, the dose optimization becomes more critical because of the electron backscattering and proximity effects. The proximity effect can extend laterally over several micrometres in many resist/substrate combinations, depending on material properties and the substrate atomic number.

Fabrication using VSB ZBA21 equipment therefore requires both precise geometrical correction and exposure-dose optimization. Geometrical correction was established first. To investigate proximity effects and evaluate the limitations of our EBL process, we designed an exposure test consisting of individual lines and line gratings. For gratings with a target period of 600 nm, we defined a minimum line-to-space (L/S) ratio of 100/500 nm, whereas for gratings with a period of 800 nm, we used an L/S ratio of 100/700 nm.

**Figure 2:**
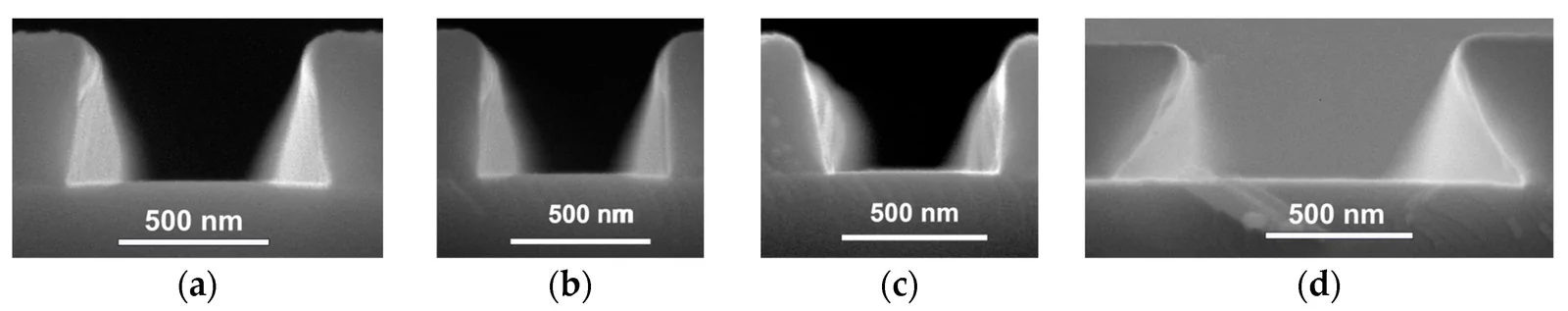
Selected cross-sectional profiles at exposure doses of: (**a**) 360 µC/cm^2^, (**b**) 400 µC/cm^2^, (**c**) 880 µC/cm^2^, and (**d**) 2120 µC/cm^2^ for a 500 nm-wide isolated line exposed in a 550 nm-thick PMMA bilayer resist.

**Figure 3:**
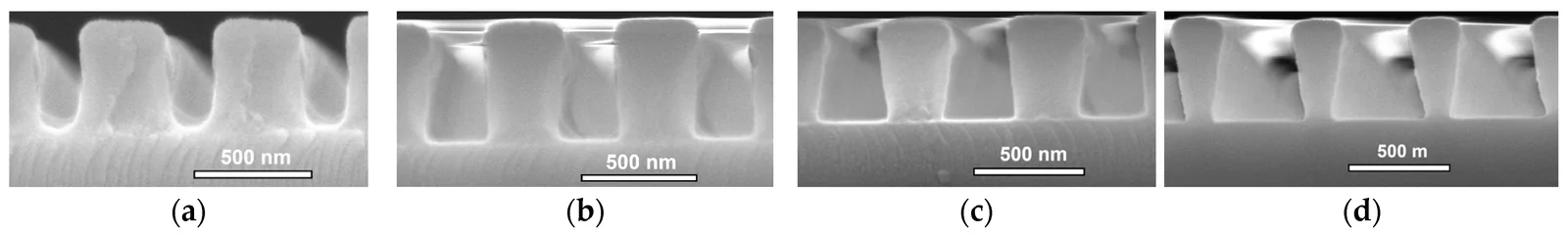
Cross-sectional profiles at exposure doses of: (**a**) 440 µC/cm^2^, (**b**) 520 µC/cm^2^, (**c**) 600 µC/cm^2^, and (**d**) 760 µC/cm^2^ for a line grating with an L/S ratio of 100/500 nm exposed in a 550 nm-thick PMMA bilayer resist.

**Figure 4:**
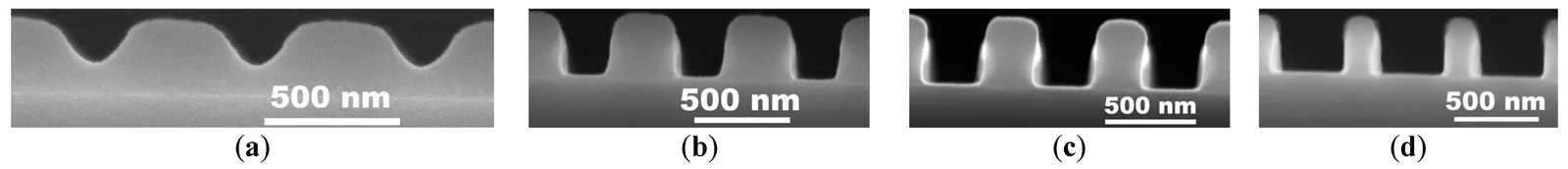
Cross-sectional profiles at exposure doses of: (**a**) 440 µC/cm^2^, (**b**) 520 µC/cm^2^, (**c**) 600 µC/cm^2^, and (**d**) 920 µC/cm^2^ for a line grating with an L/S ratio of 100/500 nm exposed in a 300 nm-thick PMMA bilayer resist.

**Figure 5:**
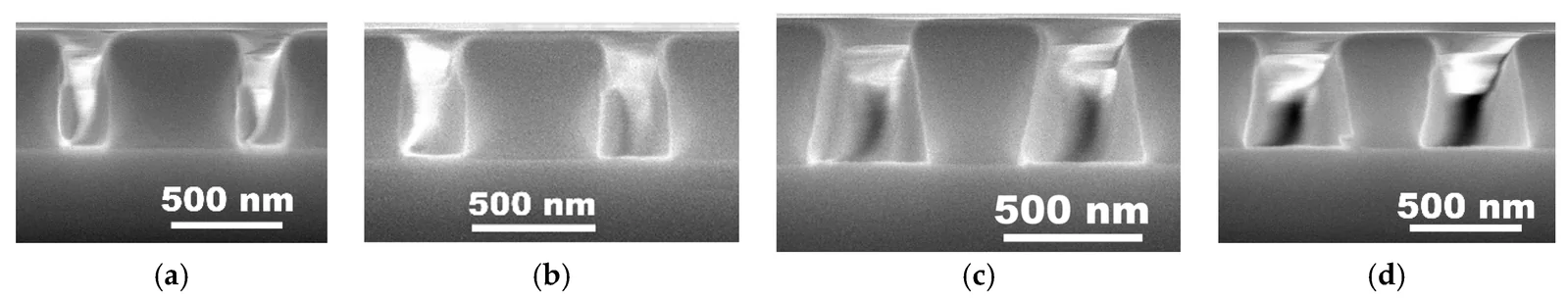
Cross-sectional profiles at exposure doses of: (**a**) 520 µC/cm^2^, (**b**) 600 µC/cm^2^, (**c**) 920 µC/cm^2^, and (**d**) 1160 µC/cm^2^ for a line grating with an L/S ratio of 100/700 nm exposed in a 550 nm-thick PMMA bilayer resist.

The dependence of the PMMA resist profile on the exposure dose is demonstrated in Figure 2, Figure 3, Figure 4 and Figure 5 for four representative lithographic patterns fabricated in a PMMA bilayer resist system with total thicknesses of 300 nm (Case 4, denoted as N4 in the figures and tables) and 550 nm (Cases 1, 3, and 5, denoted as N1, N3, and N5, respectively). The selected cases include an isolated 500 nm line and line gratings with different L/S ratios, allowing evaluation of how exposure dose and pattern density affect the developed resist profile.

Figure 2 presents the cross-sectional profiles of an isolated 500 nm line fabricated in the 550 nm-thick PMMA bilayer at exposure doses ranging from 360 µC/cm^2^ to 2120 µC/cm^2^. With increasing exposure dose, the resist profile evolves markedly, including increased lateral development and changes in sidewall geometry. At the highest dose, the developed structure broadens significantly.

Figure 3 shows the corresponding profiles for a dense line grating with an L/S ratio of 100/500 nm fabricated in the 550 nm-thick PMMA bilayer. Compared with the isolated line shown in Figure 2, the closely spaced structures show stronger profile broadening due to the proximity effect. Increasing the exposure dose further enhances the lateral development of the exposed regions and modifies the sidewall profile.

Figure 4 presents the profiles obtained for the same 100/500 nm line grating fabricated in a thinner, 300 nm-thick PMMA bilayer. Comparing these profiles with those in Figure 3 shows how resist thickness affects profile evolution. The thinner resist exhibits a different degree of lateral broadening and sidewall development, indicating that the resist thickness influences the balance between forward scattering, backscattering, and the resulting proximity effect.

Figure 5 shows the profiles of a 100/700 nm line grating fabricated in the 550 nm-thick PMMA bilayer. Compared with the denser 100/500 nm grating shown in Figure 3, the larger spacing reduces the interaction between neighbouring exposed features. Consequently, the developed profiles exhibit less pronounced proximity-induced broadening, particularly at comparable exposure doses.

Overall, Figure 2, Figure 3, Figure 4 and Figure 5 demonstrate that the developed PMMA profiles are governed by the combined effects of the exposure dose, resist thickness, and pattern density. Increasing the exposure dose generally promotes lateral development and profile broadening, while smaller line spacing enhances the proximity effect. The comparison between profiles in Figure 3 and Figure 5 is particularly useful for demonstrating the influence of pattern density, whereas the comparison between profiles in Figure 3 and Figure 4 highlights the role of resist thickness.

The experimental results show that the developed PMMA profiles depend strongly on exposure dose, resist depth, bilayer thickness, and pattern density. In contrast to the conventional EBL evaluation based primarily on the top-surface critical dimension, the cross-sectional measurements reveal substantial linewidth variations along the resist thickness. These variations suggest that exposure-dose optimization should consider the full resist profile.

Liu et al. [23] reported similar challenges in dose optimization when studying EBL on insulating substrates such as SiO_2_ and Si_3_N_4_. They found that localized charging leads to beam deflection and inconsistent feature sizes, creating very narrow exposure-dose windows. In our experiments, the underlying conductive Si substrate minimizes substrate charging. A full cross-sectional analysis is necessary to properly control the final dimensions. The experimental profiles show a systematic change in sidewall slope as exposure dose increases. At lower doses, the developed linewidth at the lower part of the resist can differ significantly from that at the upper surface, whereas at higher doses the relative widths change and the sidewall may become increasingly tapered or broadened.

A pronounced influence of the bottom 495 PMMA A6 layer thickness is observed when comparing structures with otherwise comparable pattern geometry. Despite the identical nominal pattern geometry, different exposure doses are required to obtain near-vertical profiles. This behaviour indicates that the vertical resist profile depends not only on total resist thickness but also on the relative thickness of the two PMMA layers. The larger contribution of the more sensitive bottom A6 layer in the 550 nm bilayer promotes stronger development in the lower part of the resist. When this layer is thinner, a higher exposure dose is required to obtain a comparable balance between the upper and lower regions of the developed profile.

Comparing profiles in single-line structures and gratings further highlights the difference between isolated and pattern-dependent exposure conditions. For the single lines with LW = 500 nm, the developed profiles are primarily influenced by the electron dose, resist thickness, and electron scattering within the resist/substrate system. In the line gratings, however, the additional exposure from neighbouring lines contributes to the local deposited energy.

### 3.2. Simulation Results

#### 3.2.1. Statistical Approach

**Figure 6:**
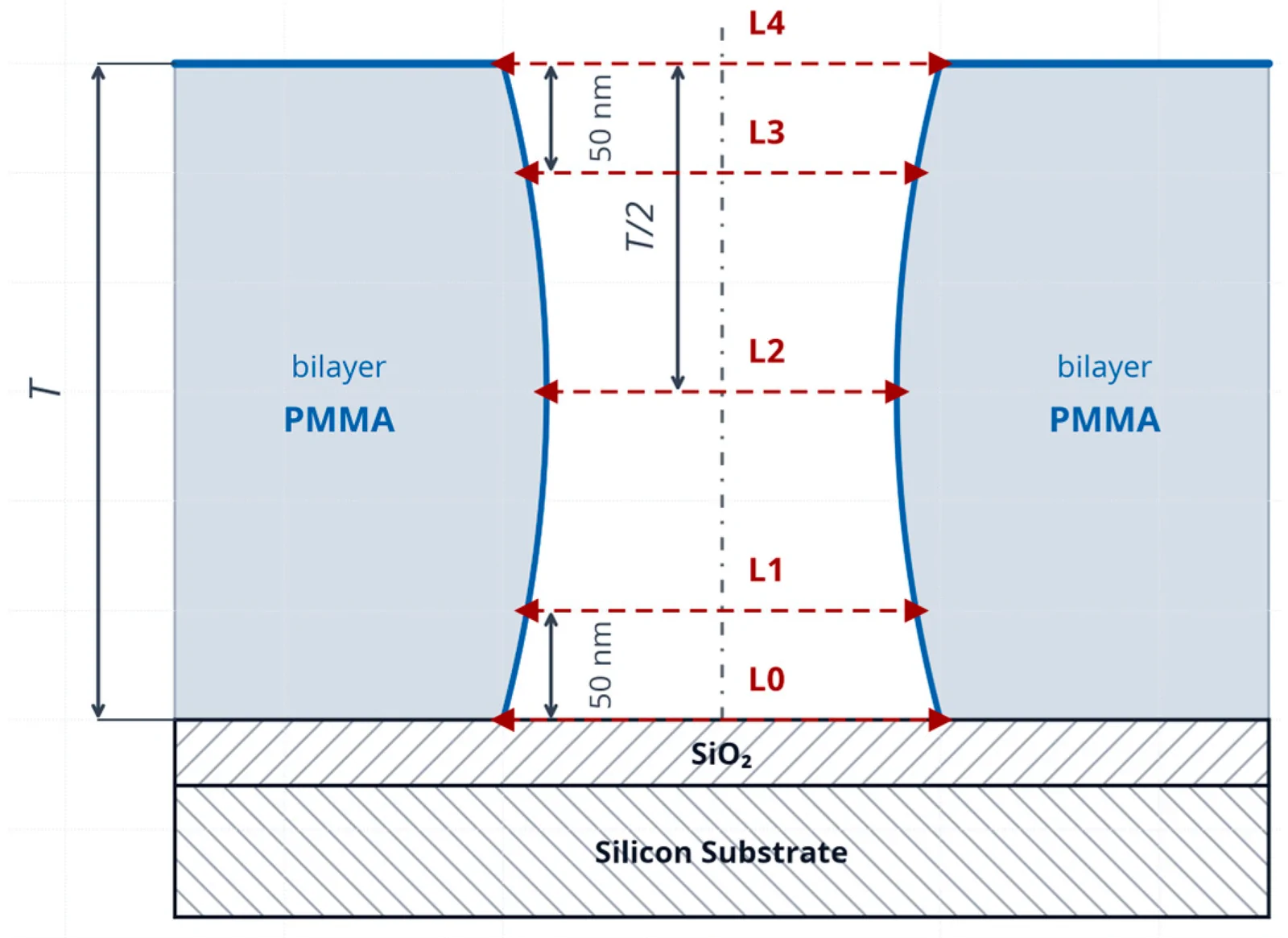
Details of the developed profile geometry for the studied structures in bilayer PMMA.

A statistical approach is applied, and equations (polynomial functions with independent variables up to the third degree) for fitting the developed bilayer linewidth Y_LW_ as a function of the exposure dose and bilayer resist depth, based on the measured data at different levels (depths—L0, L1, L2, L3, and L4) along the PMMA bilayer (Figure 1 and Figure 6), are evaluated.

The lithography process parameters—the dose of exposure (z_1_) and the resist depth (z_2_)—are studied in coded form: $x_{1}=\frac{2z_{1}-\left(D_{\max}+D_{\min}\right)}{D_{\max}-D_{\min}}$ and $x_{2}=\frac{2z_{2}-T}{T}$, where *D_max_* and *D_min_* are the maximum and minimum values of the exposure dose, and *T* is the value of the bilayer resist thickness for the studied cases, respectively. The ranges of variation in the two parameters—the exposure dose (*D*) and the bilayer PMMA resist thickness (*T*) for the investigated five test structures are mentioned above. The coded parameters (x_1_ and x_2_) vary in the range [−1, 1]. This normalization allows the statistical models to evaluate the relative weight of the dose and resist depth parameters on the profile shapes symmetrically.

The modelling equations for the linewidth Y_LW_ are a polynomial function with independent variables up to the third degree ($x_{1},x_{2},x_{1}^{2},x_{2}^{2},x_{1}x_{2},x_{1}x_{2}^{2},x_{1}^{2}x_{2}$) and the general form of the function is:

(1) $$Y_{\mathrm{LW}}(x_{1},x_{2})=a+bx_{1}+cx_{2}+dx_{1}^{2}+ex_{2}^{2}+fx_{1}x_{2}+gx_{1}x_{2}^{2}+hx_{1}^{2}x_{2}$$

Table 1 presents the evaluated equations’ coefficients for the developed linewidth Y_LW_ of the bilayer PMMA resists as functions of the dose of exposure (*x*_1_) and resist depth (*x*_2_) for the five investigated test structures (Cases 1–5).

Table 2 summarizes the goodness-of-fit and cross-validation statistics for the five empirical models (N1–N5). All models are statistically significant with F-values ranging from 39.48 (N4) to 153.54 (N1). The fitted coefficient of determination R^2^ ranges from 89.16% (N4) to 96.14% (N1), while the adjusted R^2^_adj_ ranges from 86.90% (N4) to 95.51% (N1). The variability in R^2^_pred_ and RMSE_CV_ across the five cases should be considered for the estimation of the predictive accuracy of the models. The values of the cross-validated coefficient of determination R^2^_pred_ are lower than the values of R^2^ and R^2^**_adj_** in all cases, ranging from 82.22% (N5) to 94.47% (N1). The difference between R^2^ and R^2^_pred_ values, which quantifies the degree of overfitting, is smallest for N1 (1.67 percentage points) and largest for N5 (9.83 percentage points). The residual standard error (RSE) ranges from 25.83 nm (N3) to 44.50 nm (N5), while the cross-validated RMSE_CV_ values range from 31.57 nm (N3) to 44.59 nm (N5). Case N5, despite its relatively high fitted R^2^ (92.04%), shows the largest discrepancy between fitted and cross-validated performance (9.83 percentage points), suggesting the model is over-parameterized relative to the available data. This is consistent with its large residual standard error (RSE = 44.50 nm), which is the highest among the five models. The ratio n/k of the observations n (Table 2) to the number of the regression model parameters k (Table 1) ranges from 5.0 (N3 and N4) to 9.1 (N2).

The chosen structures of the regression models presented in Table 1 are selected after consideration of the significance of the corresponding regression coefficients, change in R^2^ and R^2^_adj_, the F-distribution value of the variance ratio, as well as their influence on the RSE, R^2^_pred_, RMSE_CV_, and $\sigma_{\mathrm{error}}^{2}$.

**Figure 7:**
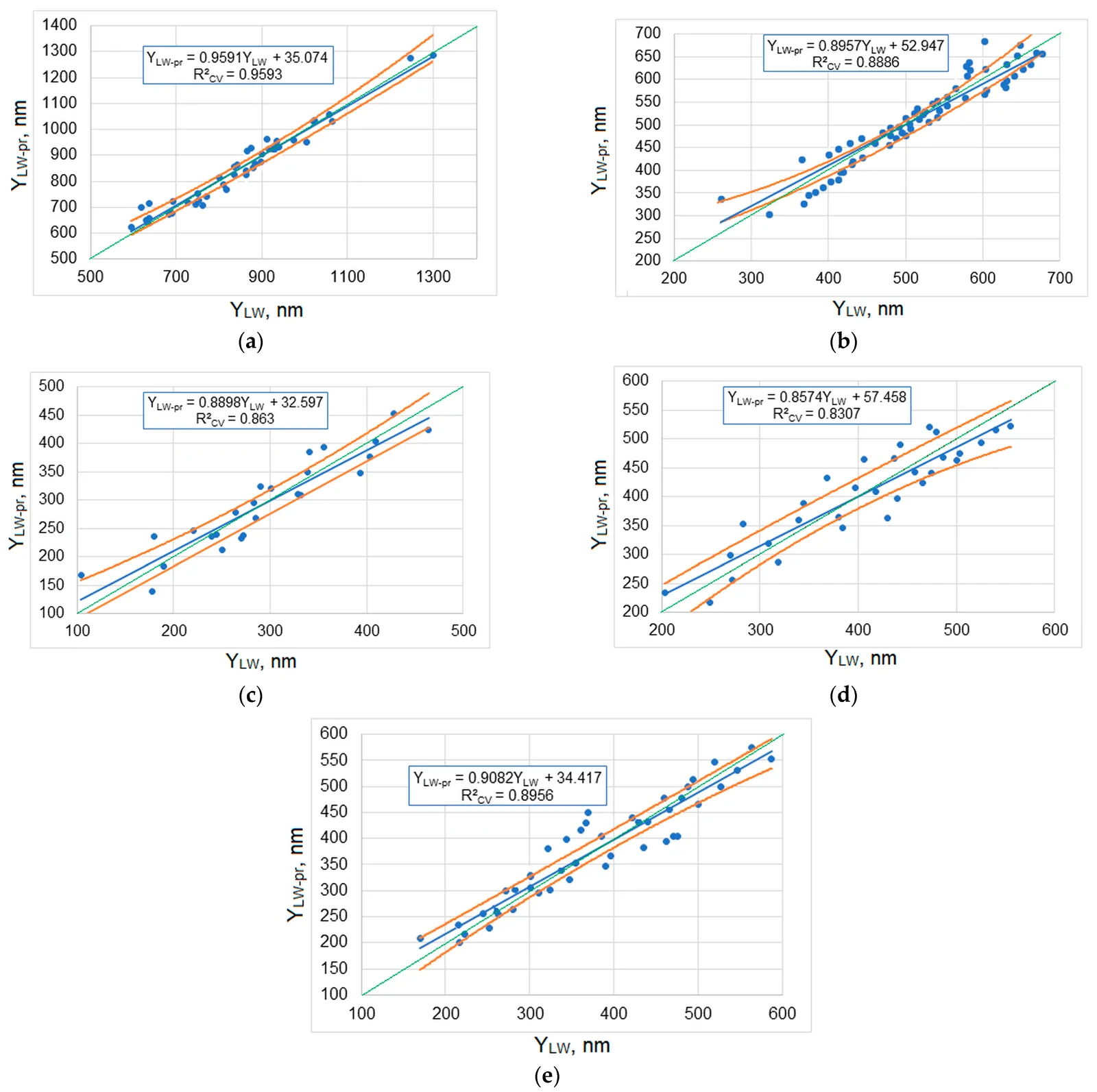
Cross-validation plots (measured Y_LW_ vs. LOO predicted Y_LW-pr_ values) for estimated empirical Y_LW_ models: (**a**) case N1, (**b**) case N2, (**c**) case N3, (**d**) case N4, and (**e**) case N5. The blue lines are the linear regression of LOO-predicted Y_LW-pr_ vs. the measured values Y_LW_; the green lines are the ideal Y_LW-pr_ = Y_LW_ lines. The orange curves show the estimated 95% confidence interval for the predicted mean Y_LW_ values.

Figure 7 presents the leave-one-out (LOO) cross-validation plots (measured YLW vs. LOO-predicted values) for the five estimated empirical models (Cases N1–N5). We obtained the LOO predictions by excluding each observation in turn, so each point represents a true out-of-sample prediction. The cross-validation coefficient of determination R^2^_CV_ (Figure 7) ranges from 83.07% (Case N4) to 95.93% (Case N1), indicating that the models explain between 83% and 96% of the variability in new observations. For all cases, the regression slope is below unity (0.857–0.959, Figure 7) and the intercept is positive (+32.6 to +57.5, Figure 7), revealing a slight systematic underestimation of high values and overestimation of low values. Cases N1 and N2 show the tightest agreement with the diagonal. Overall, the cross-validation results confirm that the empirical models generalize reasonably well within the parameter space studied, while also highlighting cases that need further refinement. Because repeated measurements were not available, Figure 7 also presents estimated 95% confidence interval curves for the predicted mean values of Y_LW_, providing a conservative estimate of prediction uncertainty. The calculated dependencies allow estimation (prediction) of the profile geometry (shape) in the studied PMMA bilayer resist systems at a given exposure dose within the corresponding studied dose ranges.

#### 3.2.2. Profile Simulation

**Figure 8:**
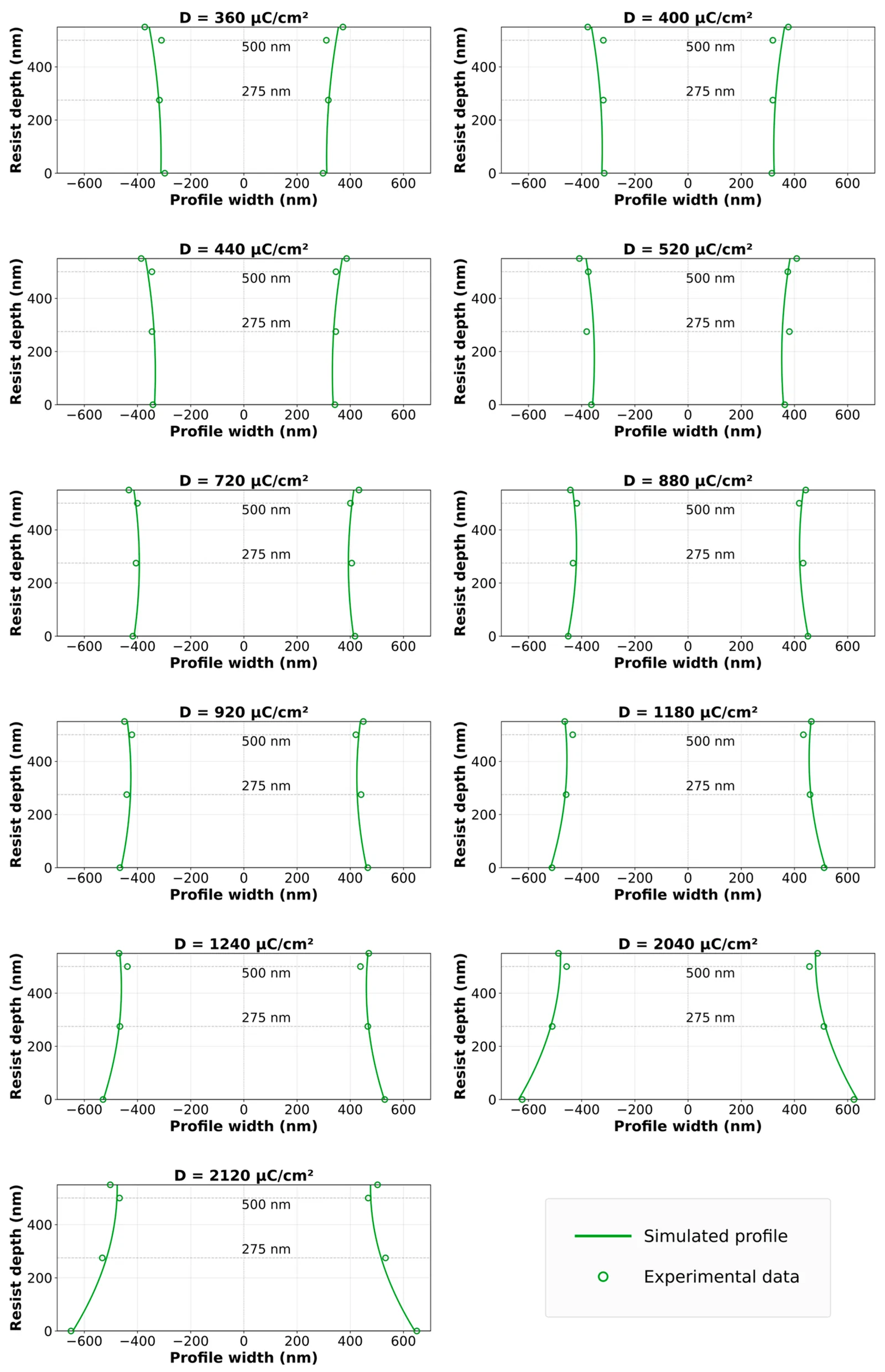
Experimental (symbols) and simulated (solid lines) developed profiles of the test structure Case 1 (a single line with LW = 500 nm) for all studied exposure doses.

**Figure 9:**
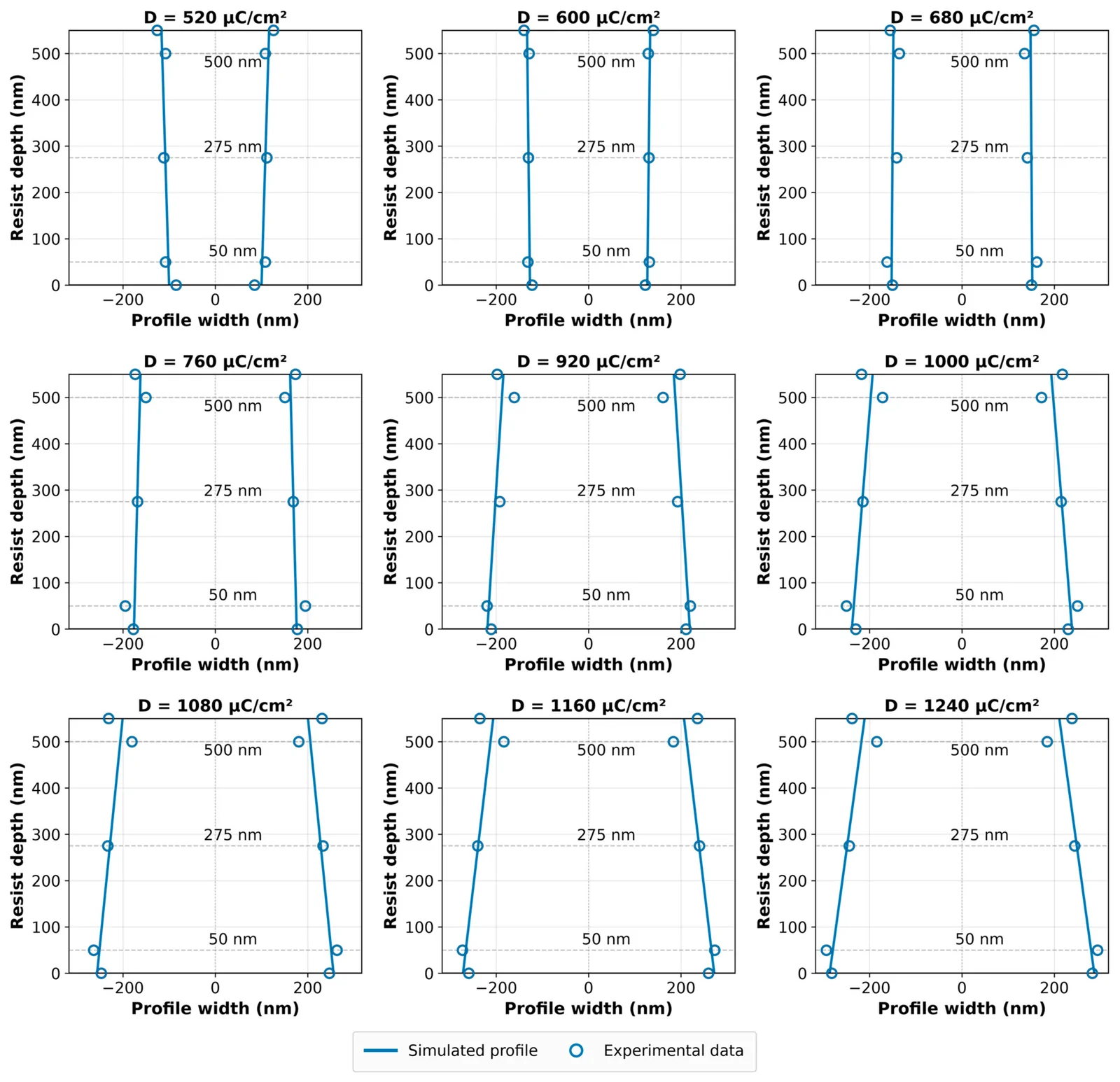
Experimental (symbols) and simulated (solid lines) developed profiles of the test structure Case 5 (a line grating with L/S = 100/700 nm) for all studied exposure doses.

Figure 8 and Figure 9 present the developed profiles of the test structures Case 1 (a single line with LW = 500 nm) and Case 5 (a line grating with L/S of 100/700 nm) in a 550 nm thick PMMA bilayer for the studied exposure doses, calculated by the model equations (Table 1), together with the experimentally measured linewidths at the levels L0-L4 (Figure 1 and Figure 6). The results trace the continuous physical evolution of the developed structures, showing the change in sidewall slope as dose increases and demonstrating very good agreement between the model and experiment for both patterns (single line and line grating).

**Figure 10:**
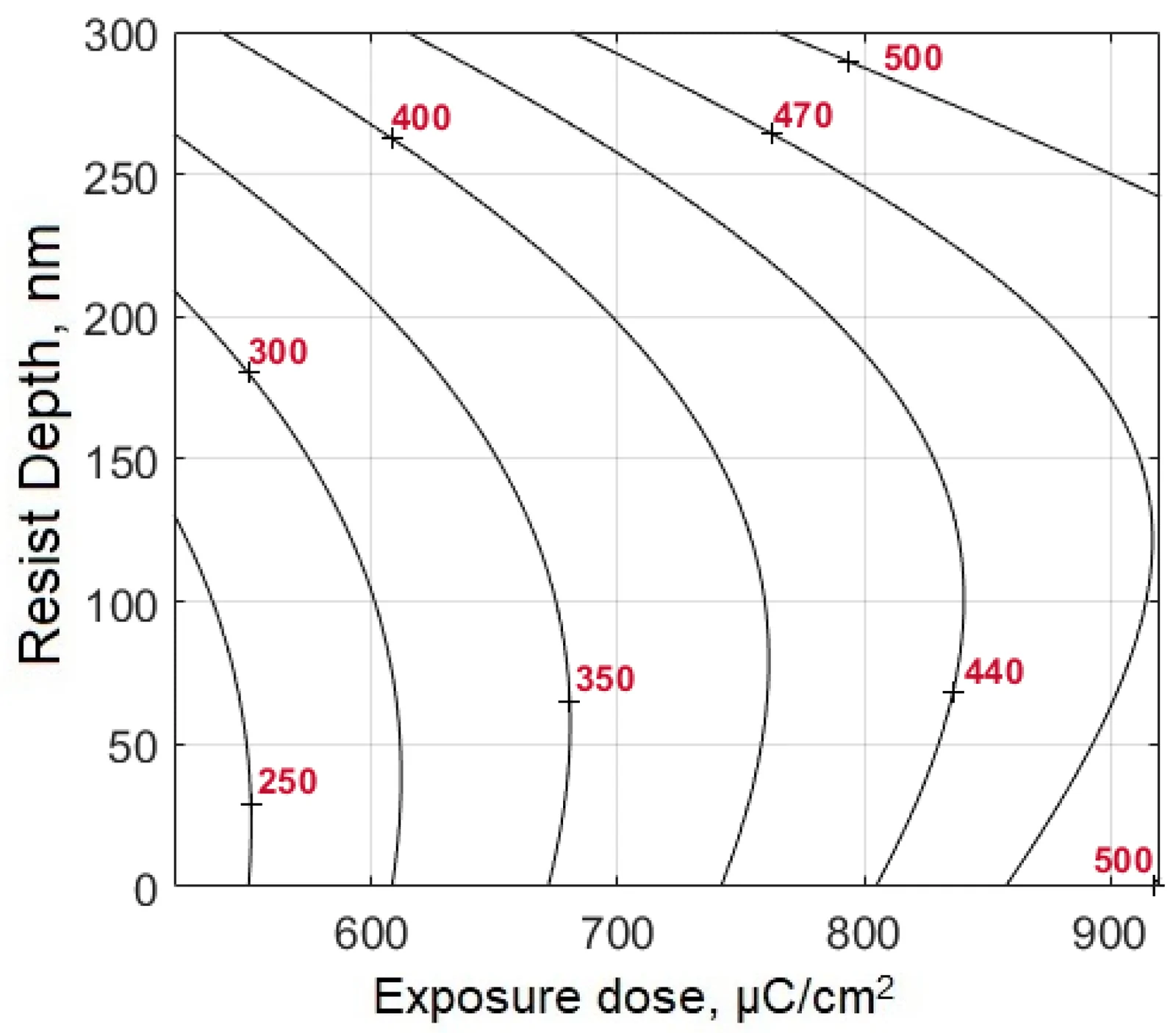
Contour plots of profile width vs. the exposure dose along the resist depth of 300 nm thick bilayer PMMA for a line grating with L/S = 100/500 nm (Case 4).

**Figure 11:**
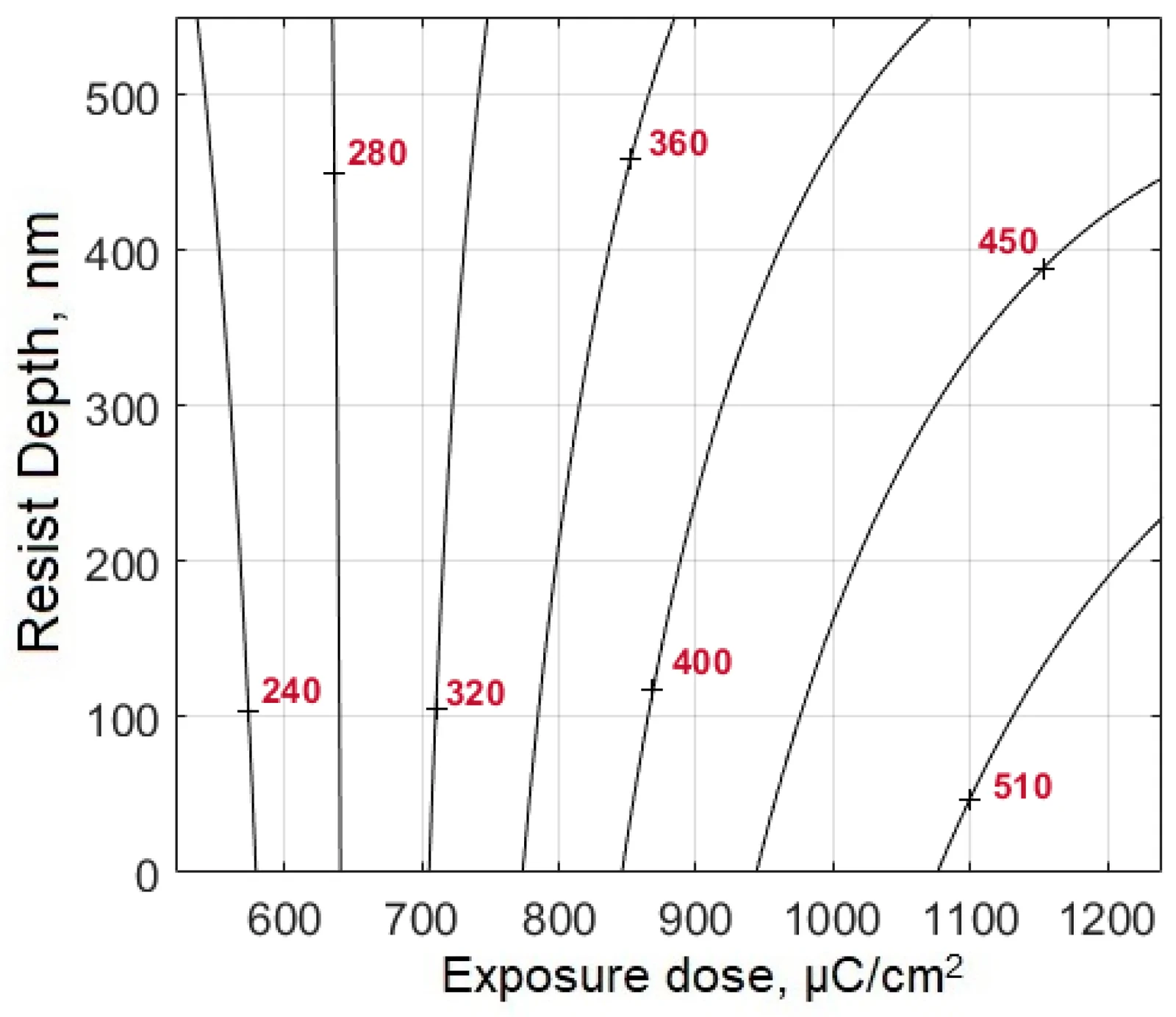
Contour plots of profile width vs. the exposure dose along the resist depth of 550 nm thick bilayer PMMA for a line grating with L/S = 100/700 nm (Case 5).

Figure 10 and Figure 11 show the calculated contour plots presenting the dependency of the developed linewidths Y_LW_ on both exposure doses and resist depth in 300 nm and 550 nm thick bilayer PMMA for the patterns corresponding to Case 4 (a line grating with L/S = 100/500 nm) and Case 5 (a line grating with L/S of 100/700 nm). These plots allow predicting the image shape of the investigated configurations in the bilayer resist systems for the corresponding dose ranges.

To estimate process repeatability, a robust engineering approach can be applied. Models for the mean values and the standard deviations of the profile linewidth can be estimated, considering: (i) the variances of the EBL process parameters—the EB exposure dose and resist depth, (ii) the influence of the process noise parameters, (iii) the estimated external variance ($\sigma_{\mathrm{error}}^{2}$, Table 2), (iv) the chosen uncertainty range factor, and (v) a normal distribution assumption. To estimate the overall output variation and account for uncertainty from external noise and process parameter variability, we implemented a Monte Carlo simulation approach. We ran 10,000 Monte Carlo simulation iterations to estimate the mean values and standard deviations of the linewidth of the PMMA bilayer resist profiles for each case (N1–N5), using Qstatlab statistical software [27]. For each iteration, random values for the noise factors were generated from their assumed distributions, and the corresponding linewidth was simulated. The standard deviation of the simulated outputs assesses process variability under the combined influence of the considered noise sources.

Two models for the mean values and the variances/standard deviations of the bilayer PMMA resist profile were estimated. The model for the mean values of the output characteristic Y_LW_ is [28,29,30]:

(2) $$\tilde{y}\left(p\right)=E\left[y\left(z\right)\right]=\eta_{m}\left(p\right)+\Theta^{T}E\left(g\right),$$

where *η_m_*(*p*) is the model of the output characteristic Y_LW_ and E(*g*) is the expected value of g. The second term accounts for the deviation caused by errors transmitted from the process parameters *p* to the output characteristic; Θ*^T^* is the vector of regression model coefficients (*η*_m_(*p*) = Θ*^T^*F); F is a matrix of known functions f of the process parameters *p*, defined by the regression model; g = h − f, where h is the vector of regressors in the regression model, considered as containing errors e (for each process parameter: z_i_ = p_i_ + e_i_).

The model for the variance is:

(3) $${\tilde{s}}^{2}=E\left(\theta^{T}\psi \psi^{T}\theta \right)+\sigma_{\varepsilon }^{2}=\theta^{T}\Psi \theta +\sigma_{\varepsilon }^{2},$$

where ψ = g − E(*g*) is determined based on the variances of each process parameter *p*—which can be calculated using the tolerance limits of the process parameters and the error covariance matrix in the presence of errors in factor levels—and Ψ = E(ψψ^T^) depends on the structure of the regression model and the experimental design; $\sigma_{\varepsilon }^{2}$ is the random error of the output characteristic. The calculation of the error covariance matrix Ψ = E(ψψ^T^) is based on certain assumptions regarding the expected value of the errors e—namely, E(e_i_) = 0 and E($e_{i}^{2}$) = $\sigma_{i}^{2}$—which can be determined using tolerance limits.

For defining the uncertainty of the process parameters—the exposure dose and resist thickness without having repeated observations, the technical specifications can be considered. Based on the literature, the measurement uncertainty of CD-SEM instruments is well characterized, and the critical dimension (CD) SEM measurement uncertainty is 1–2 nm [31,32]. We accept tolerances of *z*_2_ ± 2 nm for the variation in the resist depth definition after measurement. The exposure dose in EBL systems has several sources of uncertainty. Shot noise is a fundamental limit, and the optimal dose for CD uniformity depends on the resist sensitivity and required LER. Stochastic effects in the electron scattering and resist development can cause the actual CD to differ from the deterministic prediction. Experimental studies on PMMA resist have shown that the typical dose-induced CD variation can range from 5% to 12% of the CD, and we accepted tolerances of the exposure dose of *z*_1_ ± 6% [31,33].

**Figure 12:**
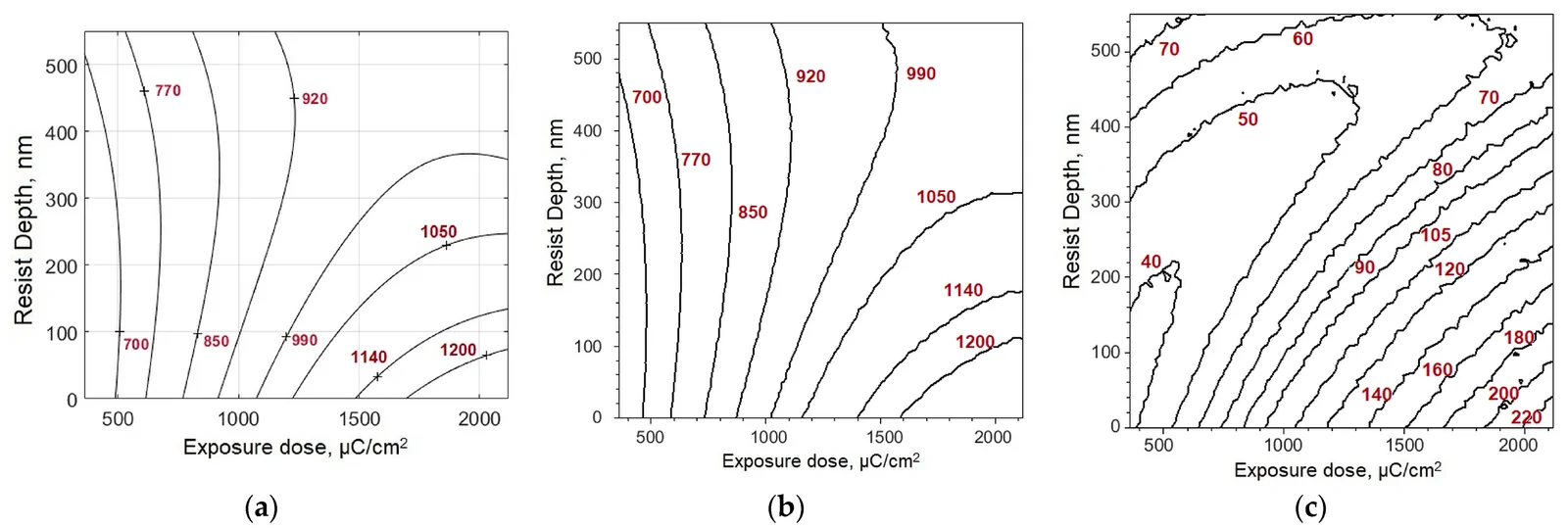
Contour plots for Case N1 (a single line with LW = 500 nm, bilayer PMMA, 550 nm total resist depth): (**a**) linewidth predicted by the regression model (Table 1) as a function of the exposure dose and resist depth; (**b**) mean linewidth obtained from the mean-value model (Monte Carlo simulation, 10,000 iterations); (**c**) standard deviation of the linewidth obtained from the variance model (Monte Carlo simulation, 10,000 iterations).

Figure 12 presents the simulated contour plots for Case N1, obtained from the two models described above. Figure 12a shows the linewidth predicted by the regression model (Table 1) as a function of the exposure dose and resist depth. Figure 12b shows the mean linewidth obtained from the mean-value model using Monte Carlo simulation with 10,000 iterations, which incorporates the propagated uncertainties of the process parameters. Figure 12c shows the corresponding standard deviation of the linewidth, calculated from the variance model, which combines the propagated parameter uncertainties with the random error of the output characteristic.

The comparison between the results shown in Figure 12a,b illustrates the effect of propagated parameter uncertainties on the mean prediction. The noises in EBL can be grouped into three categories according to their origin. Fundamental noise is dominated by shot noise, which results from statistical fluctuations in the number of electrons arriving in a given area; it is most pronounced at high resolution and low dose and directly affects CD uniformity and LER. Instrumental noise includes electron scattering and proximity effects, beam blur caused by aberrations and space-charge interactions, focus variation, and dose instability; these effects are systematic in nature and depend on the exposure system and its calibration. Material and process noise originates from the resist itself (molecular weight, nodular structure, coating uniformity) and from the development process (temperature, concentration, time, and resist swelling), all of which influence the final pattern dimensions. For uncertainty estimation, these three categories can be treated as independent contributions to the overall variance. Shot noise decreases with increasing dose, while instrumental and material noise are largely independent of dose and must be controlled through system calibration and process optimization.

The three noise categories described above are directly reflected in the results shown in Figure 12. The shot noise contribution is represented by the random error term $\sigma_{\varepsilon }^{2}$ in the variance model; because shot noise decreases as the dose increases, the standard deviation contours in Figure 12c are expected to be wider at low doses and narrower at high doses. The instrumental noise, particularly the exposure dose uncertainty of ±6% adopted here, enters the mean-value model through E(g) and the variance model through Ψ; this is why the mean linewidth in Figure 12b differs from the regression prediction in Figure 12a, and why the standard deviation in Figure 12c increases in regions where the regression model has strong nonlinear dependence on the dose. The material and process noise, represented here by the resist-depth tolerance of ±2 nm, contributes to Ψ through the depth parameter z_2_; this is reflected in the vertical gradient of the standard deviation contours in Figure 12c. Comparing Figure 12a,b therefore provides a direct visualization of the systematic shift caused by external noise, while Figure 12c quantifies the total propagated uncertainty.

The choice of the optimal work regime for EBL can involve the standard deviation minimization as an additional criterion.

#### 3.2.3. Side-by-Side Profile Comparisons

**Figure 13:**
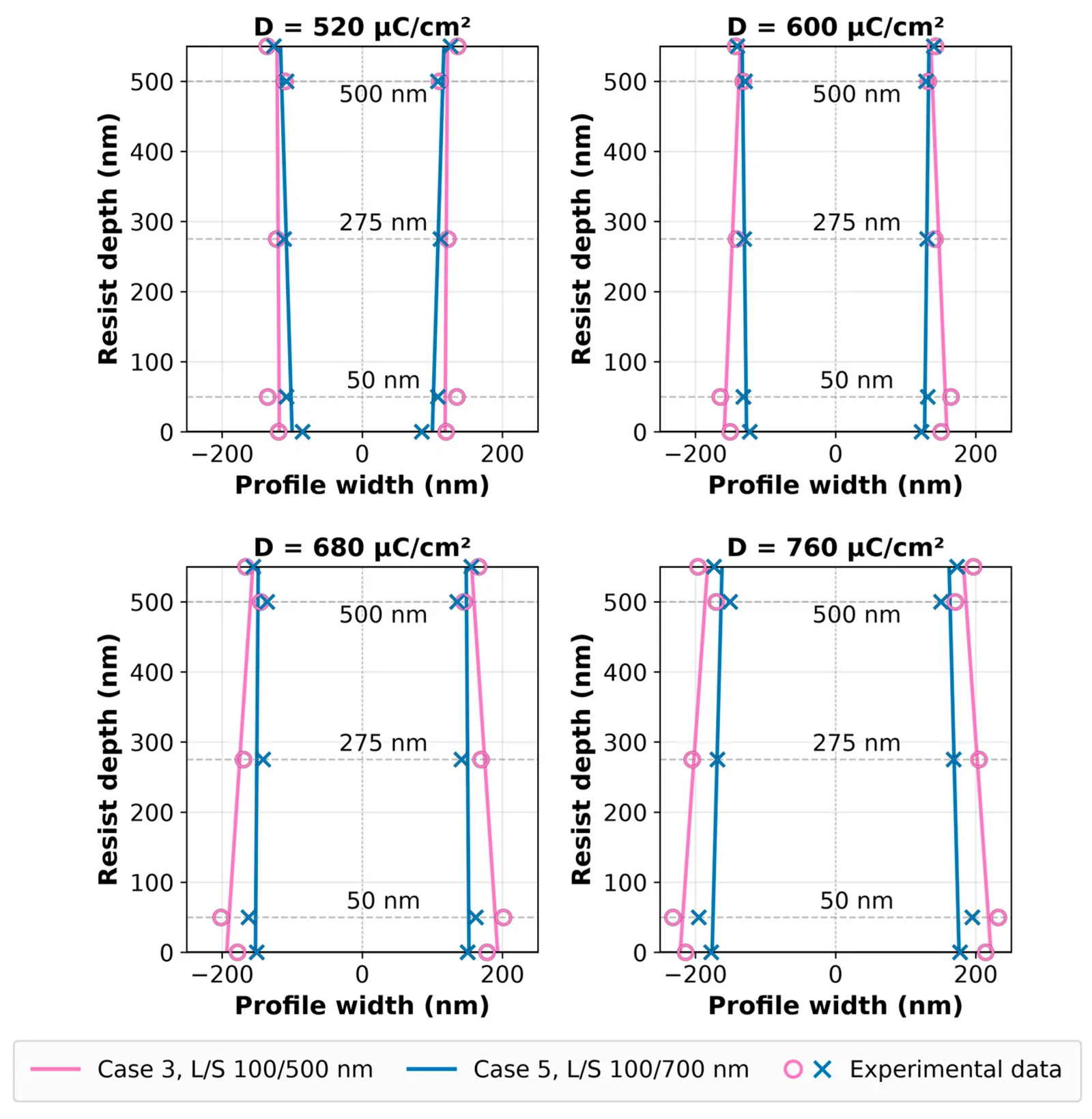
Comparison between experimental (markers) and simulated (solid lines) developed profiles for the test structures Case 3 (a grating with L/S = 100/500 nm) and Case 5 (a grating with L/S = 100/700 nm) in 550 nm thick bilayer PMMA at exposure doses of 520, 600, 680, and 760 µC/cm^2^.

Figure 13 compares experimental and simulated resist profiles for the test structures in Case 3 (grating with L/S = 100/500 nm) and Case 5 (grating with L/S = 100/700 nm) in 550 nm thick bilayer PMMA at equivalent exposure doses. The solid lines are the profiles calculated by the model equations (Table 1), and the symbols denote the experimentally measured linewidths at levels L0-L4. A very good agreement is observed between experimental and simulation results. This side-by-side representation explicitly reveals the developed line broadening caused by the proximity effect at different line spacings, showing how the denser Case 3 grating develops wider profiles (increased distortion) than the looser Case 5 grating at the same doses.

**Figure 14:**
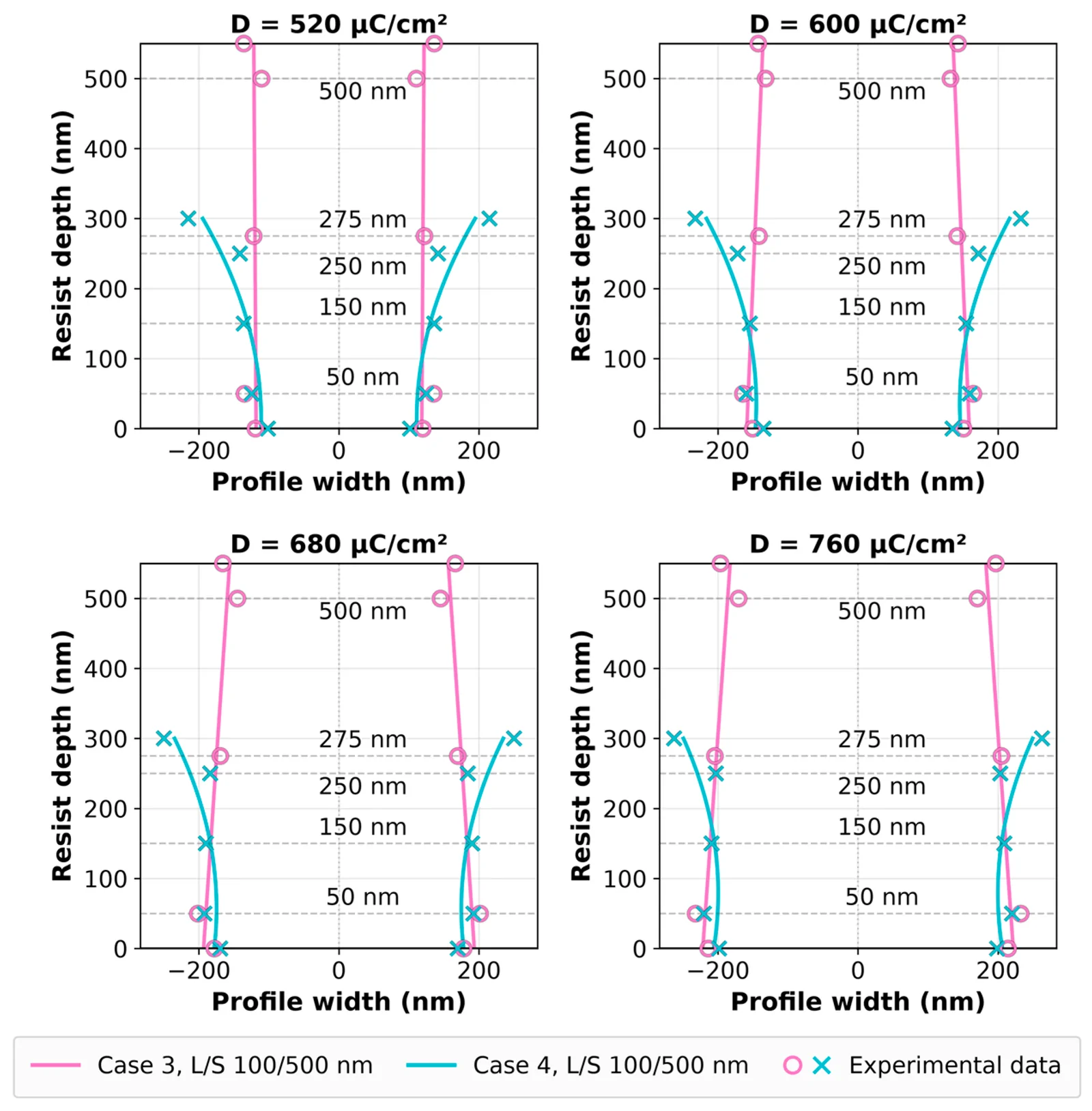
Comparison between experimental (markers) and simulated (solid lines) developed profiles for the dense grating test structures (with L/S = 100/500 nm) in bilayer PMMA systems with thicknesses of 550 nm (Case 3) and 300 nm (Case 4) at exposure doses in the range of 520–760 µC/cm^2^.

A comparison between experimental profiles and simulated ones for the dense grating test structures with an L/S ratio of 100/500 nm in different bilayer PMMA systems—Case 3 (100 nm 950 PMMA A2/450 nm 495 PMMA A6) and Case 4 (100 nm 950 PMMA A2/200 nm 495 PMMA A6) is shown in Figure 14. The solid lines are the profiles calculated by the model equations (Table 1), and the symbols denote the experimentally measured linewidths at the levels L0–L4. This visualization explicitly captures the geometric shape variations driven by forward e-beam scattering (proximity effect) and the thickness of the more sensitive bottom PMMA A6 resist layer, revealing their influence on developed-image formation and the need to use different exposure doses to form desired profiles in both bilayer resist systems.

**Figure 15:**
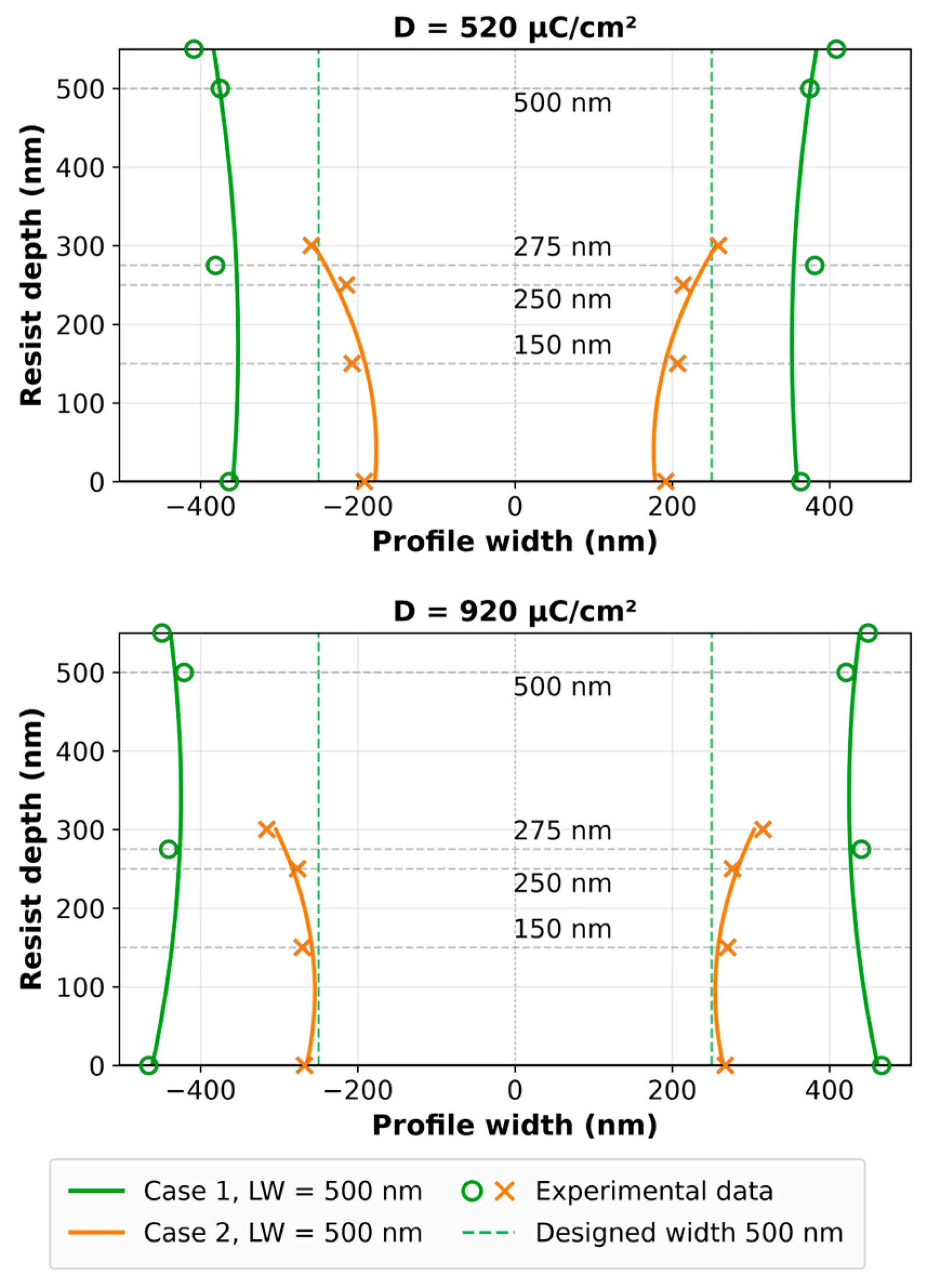
Comparison between experimental (symbols) and simulated developed profiles for a single line with LW = 500 nm exposed in 550 nm (Case 1) and 300 nm (Case 2) thick PMMA bilayers.

These influences of the internal proximity effect and the thickness of the more sensitive resist layer are also demonstrated in Figure 15 when exposing a single line (with LW = 500 nm) in the two different bilayer materials—in Case 1, the thickness of the more sensitive 495 PMMA A6 resist is 450 nm, while its thickness is 200 nm in Case 2.

#### 3.2.4. Optimization Criteria

To achieve vertical image sidewalls in the studied bilayer resist systems and configurations the following four criteria are proposed—the differences between the widths of the developed line (LW) at different depths along the bilayer resist systems: d_i_ = 0, for i = 1…4, where d_1_ = LW(L4) − LW(L0), d_2_ = LW(L2) − LW(L0), d_3_ = LW(L3) − LW(L0), and d_4_ = LW(L3) − LW(L1) (Figure 1 and Figure 6).

Based on the experimentally measured data, the corresponding modelled equations for the proposed criteria as third-degree polynomials of the exposure dose (*x*_1_) are evaluated, and the general form across all investigated test structures is:

(4) $$d_{i,j}=k_{0,\mathrm{ij}}+k_{1,\mathrm{ij}}x_{1,j}+k_{2,\mathrm{ij}}x_{1,j}^{2}+k_{3,\mathrm{ij}}x_{1,j}^{3}$$

where the index i = 1…4 denotes the criterion (d_1_–d_4_), and j = 1,2,3,4,5 denotes the investigated test structure (Case 1–Case 5). The criterion d_4_ is evaluated only for the gratings (Cases 3, 4, 5), as the level L1 is not measured for the single lines (Cases 1, 2).

Table 3 presents the evaluated coefficients *k_i_,_j_* for the criteria of Equation (4), together with the coefficients of determination R^2^; dashes denote terms not retained in the respective fit. The R^2^ values (>0.91 for all optimization criteria and studied structures except Case 2 for *d*_1_ and *d*_3_) reveal minimal errors for the proposed modelled equations.

The optimal EBL process modes according to the optimization criteria *d_i_* are reached at the exposure doses (*D*) for which *d_i_* = 0, i = 1, 2, 3, 4. Table 4 summarizes the predicted optimal dose values—the roots of the evaluated equations *d_i_*(x_1_) = 0, converted to real dose values for the four optimization criteria and the five investigated test structures. These roots represent the dose values at which the developed profiles have the closest-to-vertical sidewalls between the two levels along the resist, as defined by the criterion. For example, when applying criterion *d*_1_ (*d*_1_ = LW(L4) − LW(L0)) this is the section of the profile between levels L0 and L4, while for criterion *d*_4_ (*d*_4_ = LW(L3) − LW(L1))—between levels L1 and L3 along the resist depth (Figure 1 and Figure 6).

The summarized values (Table 4) clearly quantify the proximity effect (both internal and external for the line gratings) and the influence of the thickness of the more sensitive 495 PMMA A6 resist across the five cases studied. The results show: (i) lower optimal doses for structures containing a thicker layer of 495 PMMA A6 resist—when comparing the two single lines (Case 1 vs. Case 2) and when comparing the three line gratings (Cases 3 and 5 vs. Case 4); (ii) lower optimal doses for the denser structure of parallel lines—Case 3 compared to Case 5 across all criteria (*d*_1_–*d*_4_).

**Figure 16:**
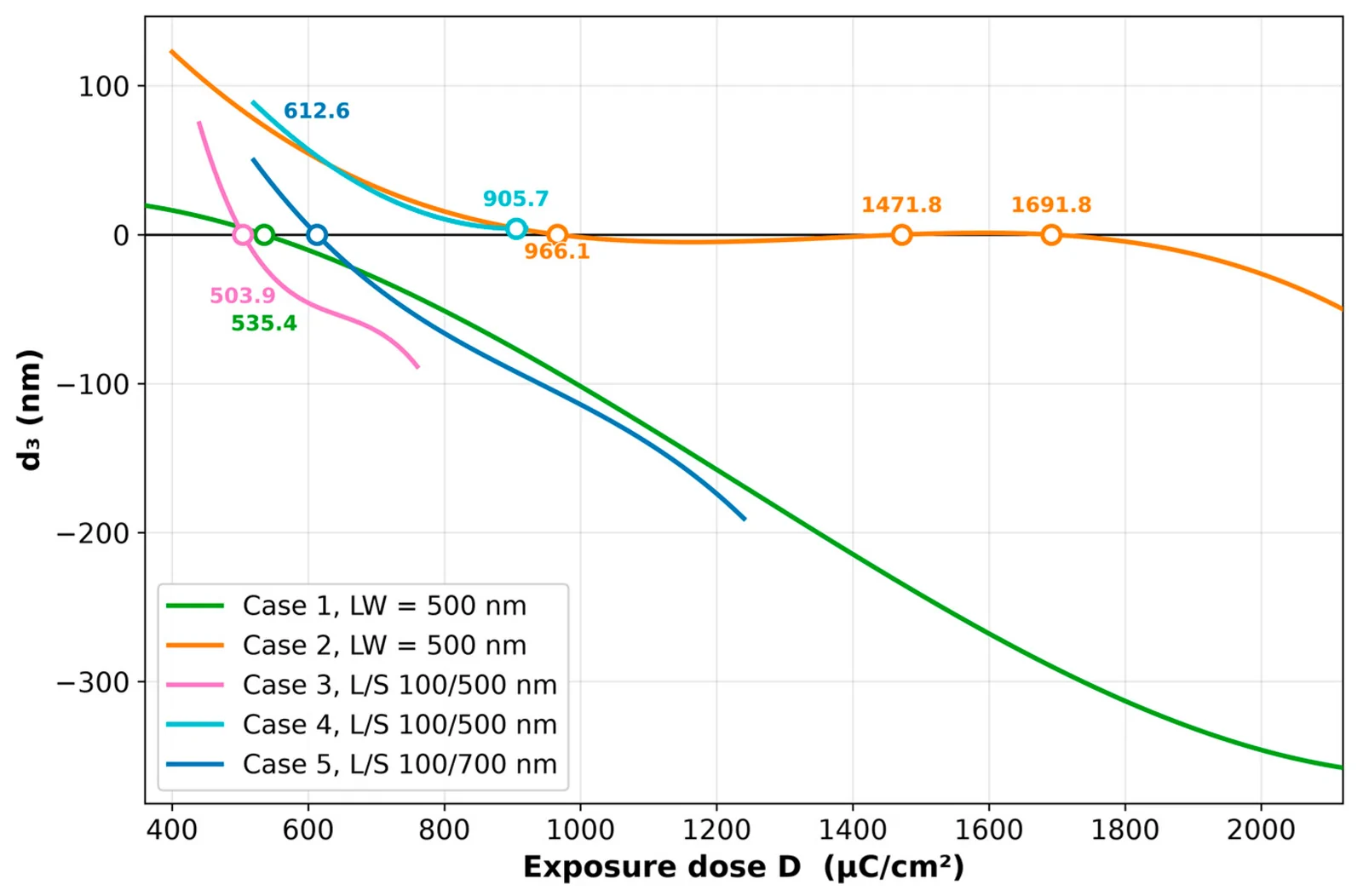
Dependence of the value of the criterion d_3_ on the exposure dose for the studied test structures (Cases 1–5) in bilayer PMMA systems; markers denote the predicted optimal doses.

**Figure 17:**
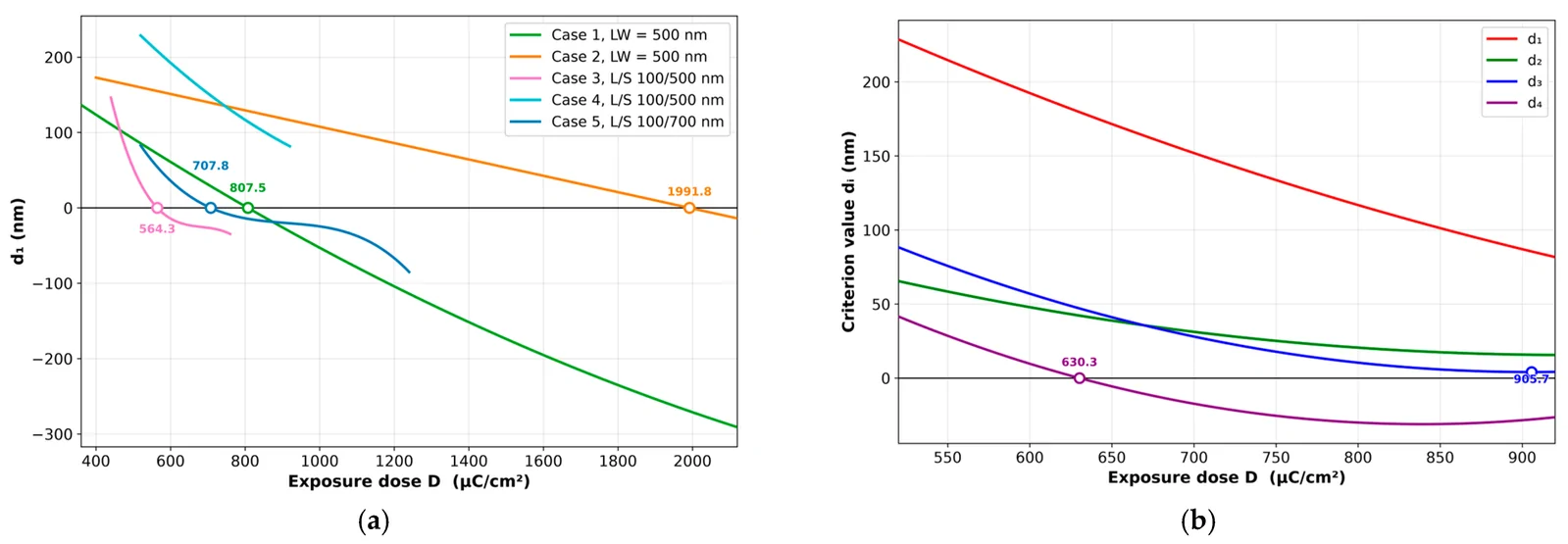
Dependences of the optimization criteria on the exposure dose for: (**a**) criterion d_1_ for the studied test structures (Cases 1–5); markers denote the predicted optimal doses; (**b**) criteria *d*_1_*–d*_4_ for the test structure Case 4 (300 nm thick bilayer PMMA, line grating with L/S = 100/500 nm).

Figure 16 and Figure 17 show the dependence of the optimization criterion values (d_3_ and d_1_) on exposure dose for the studied test structures, calculated using the evaluated equations (Equation (4), Table 3). These curves show the sensitivity of the sidewall shape (change in sidewall slope) to exposure dose, transitioning from positive values at low doses to negative values at higher doses. The optimal EBL process modes, where the desired verticality is achieved, are reached at the doses for which *d_i_* = 0 (i = 1,2,3,4).

For Case 2 (a single line with LW = 500 nm in a 300 nm thick bilayer PMMA), based on the d_3_ = 0 criterion, the optimal doses are 966.1, 1471.8, and 1691.8 μC/cm^2^, respectively (Figure 16). This defines an optimal dose range from ~960 to ~1700 μC/cm^2^, where the sidewalls of the developed images are closest to vertical between levels L0 and L3 along the resist depth.

A negative value of the *d*_3_ criterion for a given dose *D* indicates that the profile is wider at the resist/substrate interface (at L0 level) compared to its width at level L3, whereas a positive *d*_3_ value indicates a narrower profile at L0 compared to level L3 for the corresponding dose (Figure 16).

For cases where a given criterion does not reach zero within the studied dose range, the doses showing the minimum deviation from zero are identified as the optimal doses. As shown in Figure 17, for Case 4 and criteria *d*_1_ and *d*_2_, the optimal dose is 920 μC/cm^2^.

**Figure 18:**
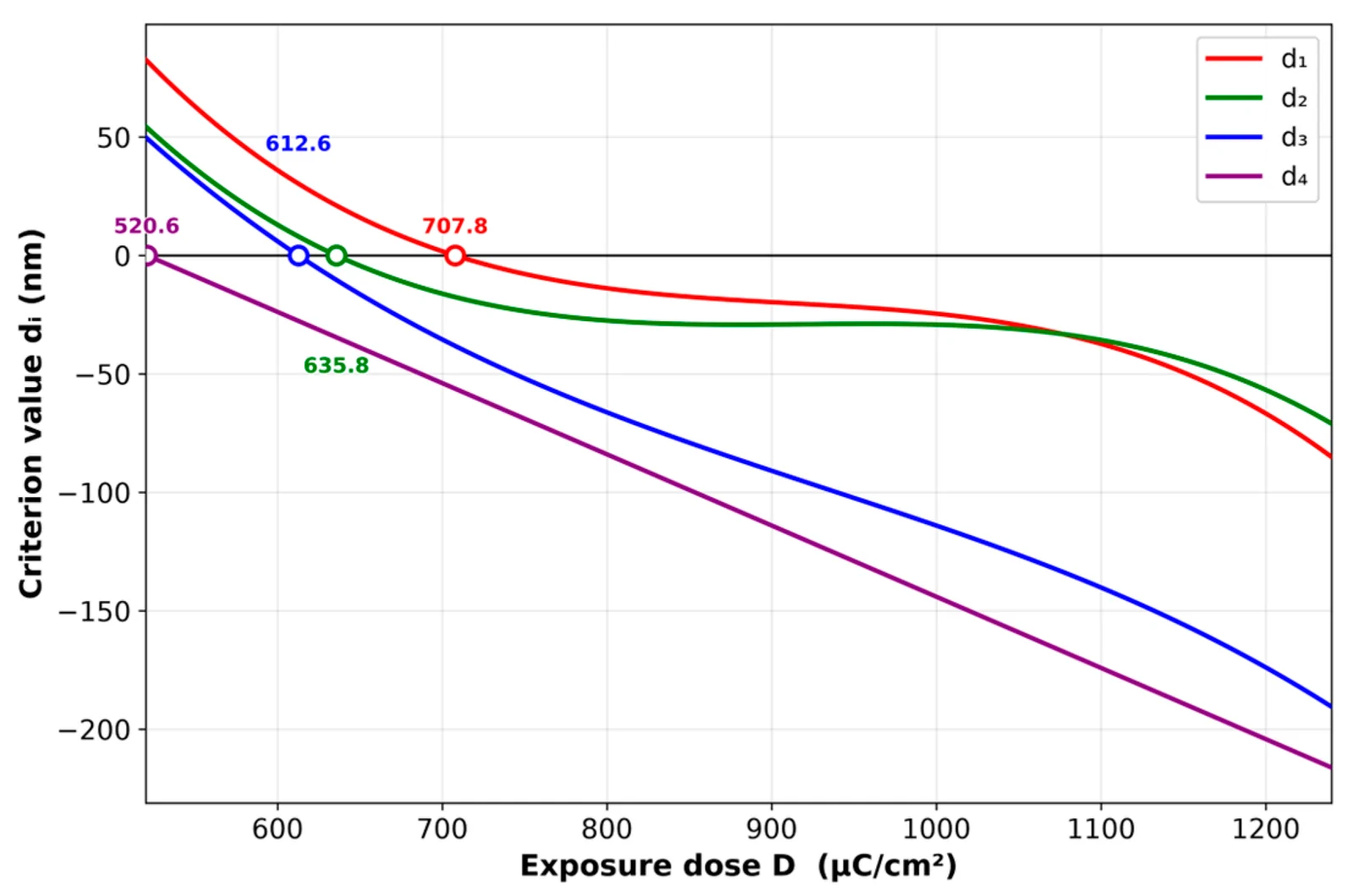
Dependence of the values of the optimization criteria *d*_1_*–d*_4_ on the exposure dose for the test structure Case 5 (550 nm thick bilayer PMMA, a line grating with L/S = 100/700 nm).

Figure 18 presents the dose dependence of the optimization criteria *d*_1_*–d*_4_ for the test structure Case 5, calculated using the regression model (Equation (4), Table 3). The curve crossings of the zero line give the optimal process doses for that structure under each criterion. Comparing the distinct zero-crossings (the optimal doses for achieving verticality in the various segments along the resist depth) reveals whether the profile shape across the whole resist depth reaches verticality simultaneously, i.e., whether these optimal doses are identical or very close in value.

**Figure 19:**
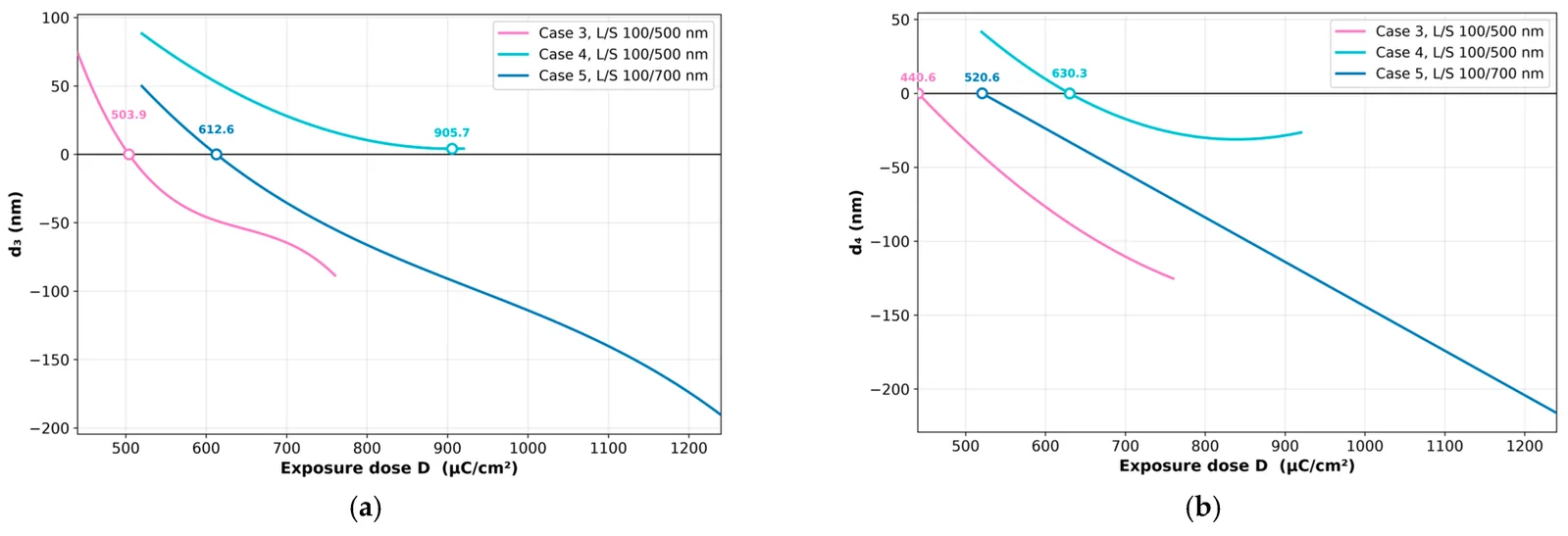
(**a**) Dependence of the value of the criterion *d*_3_ on the exposure dose for the grating test structures with L/S = 100/500 nm (Cases 3 and 4) and L/S = 100/700 nm (Case 5) in bilayer PMMA systems; markers denote the predicted optimal doses. (**b**) Dependence of the value of the criterion *d*_4_ on the exposure dose for the grating test structures with L/S = 100/500 nm (Cases 3 and 4) and L/S = 100/700 nm (Case 5) in bilayer PMMA systems; markers denote the predicted optimal doses.

Figure 19 presents the dependence of the values of criteria *d*_3_ and *d*_4_ (very important from a practical standpoint for line grating structures) on the dose of exposure for the grating test structures (Cases 3 and 4: with L/S = 100/500 nm, Case 5: with L/S = 100/700 nm) in different bilayer resist systems: 550 nm thick bilayer PMMA for Case 3 and Case 5, and 300 nm thick bilayer PMMA for Case 4. This visual comparison captures the influence of the proximity effect (including the influence of different line spacing) and bilayer resist thickness (the thickness of the more sensitive bottom PMMA A6 resist layer) on the image. It can be seen that higher optimal doses are required for Case 4 for both criteria *d*_3_ and *d*_4_, where the thickness of the more sensitive bottom PMMA A6 resist layer is less (200 nm) than in the other two Cases 3 and 5 (450 nm). Furthermore, for an identical bilayer PMMA system, the higher-density grating (Case 3) requires lower exposure doses to reach the vertical sidewalls (d_i_ = 0) than the lower-density grating (Case 5) because of the proximity effect (the cumulative influence from adjacent lines). Specifically, for Case 3, the optimal doses are 503.9 μC/cm^2^ and 440.6 μC/cm^2^ for criteria *d*_3_ and *d*_4_, respectively, whereas for Case 5, they are 612.6 μC/cm^2^ and 520.6 μC/cm^2^ for criteria *d*_3_ and *d*_4_, respectively (Figure 19).

#### 3.2.5. Optimal Profiles of Studied Structures in the Bilayer PMMA Systems According to the Proposed Optimization Criteria *d*_1_–*d*_4_

**Figure 20:**
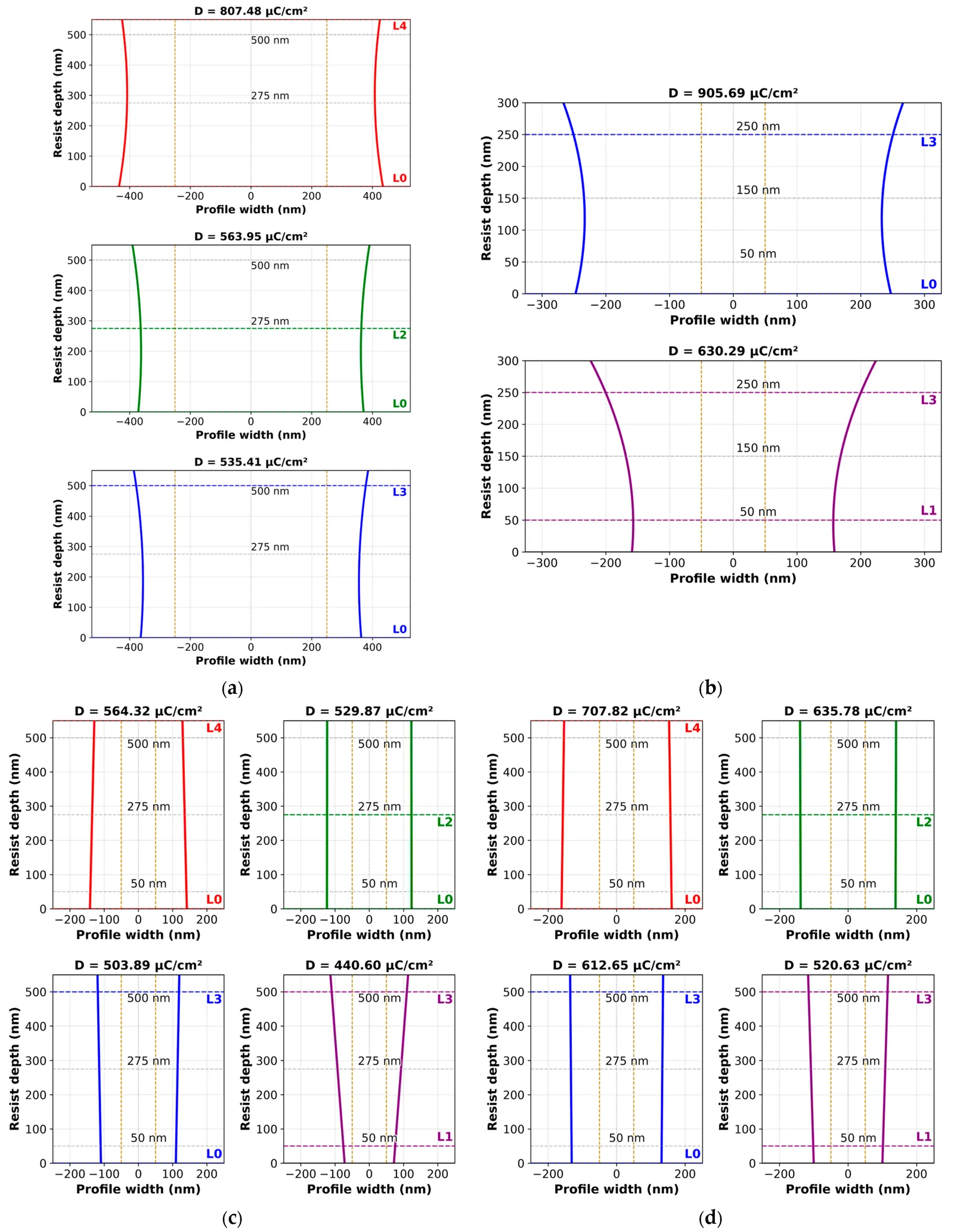

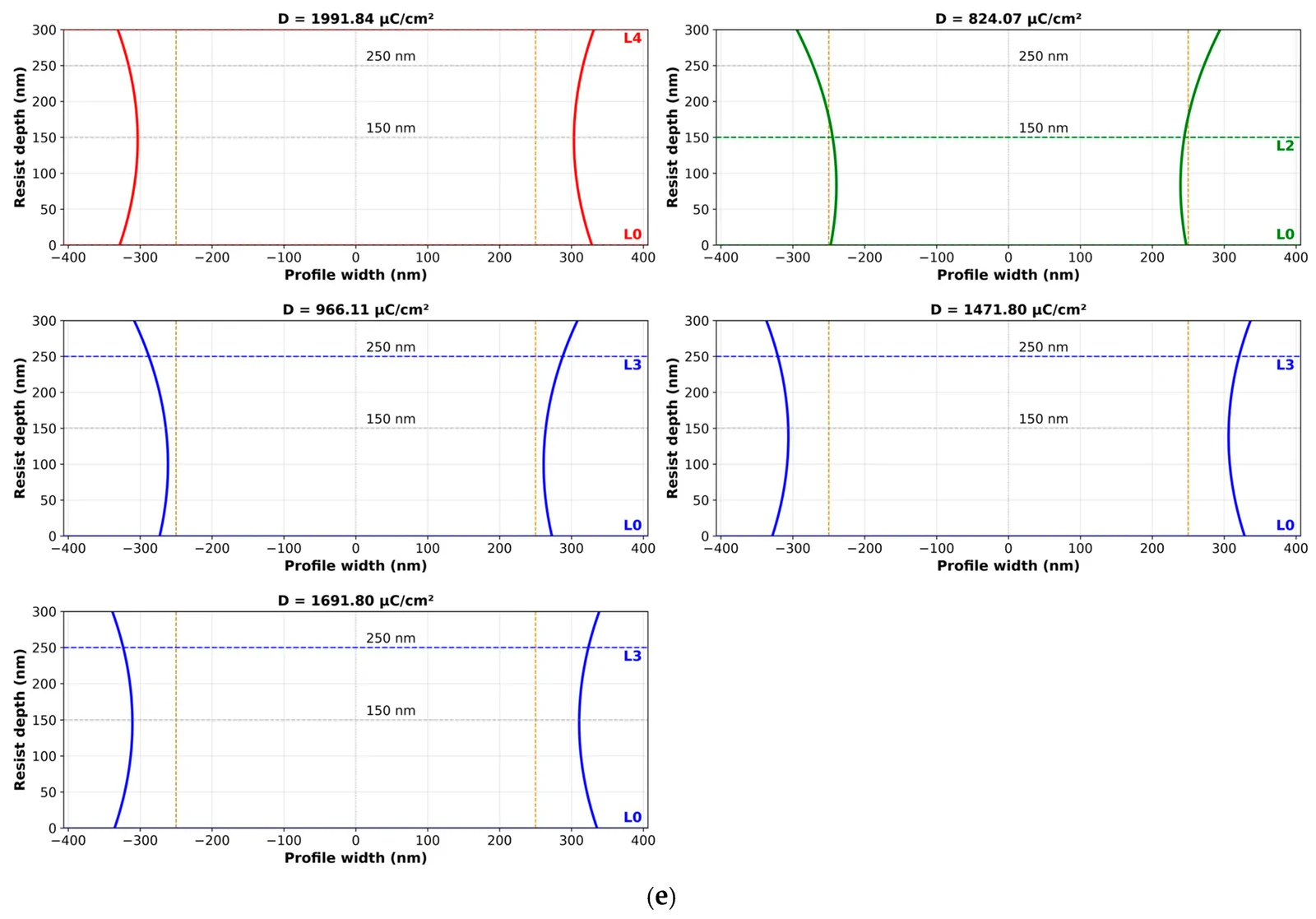
Developed profiles in the bilayer PMMA resist systems for the studied test structures (Cases 1–5) for the optimal exposure doses according to the optimization criteria *d*_1_*–d*_4_. (**a**) Case 1—a single line with LW = 500 nm in 550 nm thick bilayer PMMA. (**b**) Case 4—a line grating with L/S = 100/500 nm in 300 nm thick bilayer PMMA. (**c**) Case 3—a line grating with L/S = 100/500 nm in 550 nm thick bilayer PMMA. (**d**) Case 5—a line grating with L/S = 100/700 nm in 550 nm thick bilayer PMMA. (**e**) Case 2—a single line with LW = 500 nm in 300 nm thick bilayer PMMA.

Figure 20 presents the simulated optimal developed profiles in the studied PMMA bilayer resist systems for the line grating test structures in Cases 3, 4, 5 at the corresponding exposure doses according to the optimization criteria *d*_1_*–d*_4_ (Table 4). Each subplot corresponds to one criterion and highlights the two levels along the resist depth (associated with that criterion) between which the profile sidewall is closest to vertical.

These results (as shown in each subplot, Figure 20) make it possible to evaluate the predicted shapes and linewidths of the developed profiles along the resist depth for the corresponding optimal dose (optimization criterion) for the studied structures in the investigated bilayer PMMA systems and allow the most suitable dose for specific applications to be determined and recommended.

Results for the five investigated structures demonstrate that proximity-effect correction should not be based solely on the nominal linewidth of an individual structure. For dense line patterns, the spatial distribution of neighbouring features must also be considered because the optimum dose to achieve a desired three-dimensional profile depends on the local pattern density.

The proposed *d*_1_*–d*_4_ criteria provide a practical method for converting the measured depth-dependent linewidth data into quantitative exposure-dose optimization criteria. The results therefore support a shift from conventional CD-based optimization toward profile-based EBL optimization. Instead of defining a single target linewidth at the resist surface, the process can be optimized using a set of depth-dependent geometrical criteria. An overall optimization criterion can be defined, based on different multicriteria approaches such as the desirability function and distance function. These approaches provide additional control over the resist profile and can be particularly useful for lift-off processes, where the sidewall geometry directly influences metal deposition and pattern transfer.

## 4. Conclusions

This study presents a systematic experimental and theoretical investigation of profile evolution in bilayer PMMA resist systems for different pattern configurations exposed by 20 keV VSB EBL. The main objective was to move beyond conventional top-view linewidth analysis and establish a quantitative relationship between exposure dose, resist depth, pattern configuration, and the resulting sidewall geometry.

A methodology for empirical modelling of resist profiles and uncertainty quantification in electron beam lithography is proposed. The approach combines regression model estimation with estimation of models for mean values and standard deviations (a robust engineering approach), based on propagating process-parameter tolerances and external noise through the nonlinear process model. The models are calibrated on five experimental datasets of EBL of PMMA bilayer resists and validated by leave-one-out cross-validation, and the propagated uncertainties are evaluated by Monte Carlo simulation.

Overall, the results demonstrate that the optimum EBL dose is determined by the combined effects of exposure dose, bilayer thickness, bottom-layer thickness, and pattern density. The controlled comparisons between the investigated cases show that these parameters cannot be treated independently when specific sidewall geometry is required. The combination of cross-sectional SEM measurements, depth-dependent modelling, and the proposed (*d_i_*) optimization criteria therefore provides a useful methodology for selecting EBL process conditions to achieve controlled PMMA profiles. The proposed approach provides a quantitative empirical method for predicting, analyzing, controlling, and optimizing developed resist profiles for precise fabrication using electron beam lithography, and it can also be applied, with appropriate experimental calibration, to different resist systems, exposure dose ranges, pattern geometries, beam shot sizes, and acceleration voltages.

## Figures and Tables

**Table 1:** Developed line width models *Y_LW_*(*x*_1_,*x*_2_).

Equation Term	Coefficient	Case N1	Case N2	Case N3	Case N4	Case N5
Constant	*a*	937.37903	588.05554	294.60000	389.16844	388.36930
*x*_1_	*b*	196.28467	130.67430	109.60000	106.28571	140.24736
*x*_2_	*c*	−61.48273	17.11388	−23.05877	49.98077	−29.55100
$x_{1}^{2}$	*d*	−101.42110	−127.58650	—	−22.92411	−31.53995
$x_{2}^{2}$	*e*	57.53555	52.67046	—	45.80102	—
*x*_1_ *x*_2_	*f*	−105.19404	−43.35042	−40.48762	−33.28846	−46.08173
$x_{1}x_{2}^{2}$	*g*	29.50173	—	—	—	—
$x_{1}^{2}x_{2}$	*h*	—	30.91385	24.46605	—	—

**Table 2:** Summary of goodness-of-fit and cross-validation statistics for the line width models *Y_LW_*(*x*_1_,*x*_2_).

Parameter	Designation	Case N1	Case N2	Case N3	Case N4	Case N5
Coefficient of determination	R^2^	0.96139	0.91774	0.92622	0.8916	0.92044
Adjusted coefficient of determination	R^2^_adj_	0.95512	0.90908	0.91146	0.86901	0.91248
Cross-validated determination coefficient	R^2^_pred_	0.94474	0.88546	0.86222	0.82979	0.82220
Predicted Residual Error Sum of Squares	PRESS	57,779.41	78,608.82	24,922.36	42,393.87	89,452.17
Residual Standard Error	RSE	33.03	31.47	25.83	33.54	44.50
Cross-validated RMSE	RMSE_CV_	36.24	35.05	31.57	37.59	44.59
External variance	$\sigma_{\mathrm{error}}^{2}(\mathrm{Adj MS})$	1091.2256	990.4438	667.2875	1124.9663	1000.6497
CD-SEM experiments	n	44	64	25	30	45
Residual degrees of freedom	DF	37	57	20	24	40
F-distribution value	F	153.54	105.98	62.77	39.48	53.51

**Table 3:** Evaluated polynomial coefficients (*k_i_*_,*j*_) and corresponding coefficients of determination (*R*^2^) for the proposed profile optimization criteria (*d*_1_ − *d*_4_) calculated by Equation (4) for the investigated test structures.

Criterion (*i*)	Case (*j*)	*k*_0,*ij*_	*k*_1,*ij*_ (*x*)	*k*_2,*ij*_ (*x*^2^)	*k*_3,*ij*_ (*x*^3^)	$R_{\mathrm{ij}}^{2}$
1 (*d*_1_)	1	−113.97	−213.81	36.77	—	0.996
	2	79.45	−93.36	—	—	0.572
	3	−14.20	−45.83	70.00	−44.67	1.000
	4	144.50	−73.50	10.71	—	0.999
	5	−18.89	−15.36	17.68	−68.53	0.996
2 (*d*_2_)	1	−104.85	−136.48	—	—	0.977
	2	−14.94	—	36.20	−43.30	0.977
	3	−18.60	−11.83	52.00	−40.67	1.000
	4	28.59	−24.93	11.94	—	0.921
	5	−29.20	—	20.90	−62.73	0.975
3 (*d*_3_)	1	−169.16	−251.60	—	62.91	0.994
	2	−4.20	13.14	40.45	−99.47	0.719
	3	−45.83	−36.83	38.86	−44.67	0.990
	4	23.59	−42.07	22.66	—	0.926
	5	−86.24	−84.75	15.90	−35.47	0.997
4 (*d*_4_)	1	N/A	—	—	—	—
	2	N/A	—	—	—	—
	3	−76.74	−62.80	14.29	—	0.974
	4	−21.00	−34.00	28.57	—	0.918
	5	−108.00	−108.22	—	—	0.979

**Table 4:** Predicted optimal exposure doses for the studied Cases 1–5.

Criterion	Case N1 (μC/cm^2^)	Case N2 (μC/cm^2^)	Case N3 (μC/cm^2^)	Case N4 (μC/cm^2^)	Case N5 (μC/cm^2^)
d_1_	807.48	1991.84	564.32	Not Reached	707.82
d_2_	563.95	824.07	529.87	Not Reached	635.78
d_3_	535.41	interval [950–1700]	503.89	905.69	612.65
d_4_	N/A	N/A	440.60	630.29	520.63

## Data Availability

The original contributions presented in this study are included in the article. Further inquiries can be directed to the corresponding author.

## Abbreviations

The following abbreviations are used in this manuscript:

CD	Critical Dimension
D	Dose
EBL	Electron Beam Lithography
IPA	Isopropyl Alcohol
L/S	Line/Space
SEM	Scanning Electron Microscopy
SiO_2_	Silicon Dioxide
PMMA	Polymethyl Methacrylate
VSB	Variable Shaped Beam
